# Integrative approach for the analysis of the proteome-wide response to bismuth drugs in *Helicobacter pylori* †
†Electronic supplementary information (ESI) available: Experimental procedures, supplementary figures, identified proteomes and bioinformatics data. See DOI: 10.1039/c7sc00766c



**DOI:** 10.1039/c7sc00766c

**Published:** 2017-04-19

**Authors:** Yuchuan Wang, Ligang Hu, Feng Xu, Quan Quan, Yau-Tsz Lai, Wei Xia, Ya Yang, Yuen-Yan Chang, Xinming Yang, Zhifang Chai, Junwen Wang, Ivan K. Chu, Hongyan Li, Hongzhe Sun

**Affiliations:** a Department of Chemistry , The University of Hong Kong , Pokfulam Road , Hong Kong , P. R. China . Email: hsun@hku.hk; b School of Chemistry , Sun Yat-sen University , Guangzhou , P. R. China; c Center for Genome Sciences , The University of Hong Kong , Hong Kong , P. R. China; d CAS Key Laboratory of Nuclear Analytical Techniques , Institute of High Energy Physics , Chinese Academy of Sciences , Beijing , P. R. China; e Center for Individualized Medicine , Department of Health Sciences Research , Mayo Clinic , Scottsdale , AZ 85259 , USA; f Department of Biomedical Informatics , Arizona State University , Scottsdale , AZ 85259 , USA

## Abstract

Bismuth drugs, despite being clinically used for decades, surprisingly remain in use and effective for the treatment of *Helicobacter pylori* infection, even for resistant strains when co-administrated with antibiotics. However, the molecular mechanisms underlying the clinically sustained susceptibility of *H. pylori* to bismuth drugs remain elusive. Herein, we report that integration of in-house metalloproteomics and quantitative proteomics allows comprehensive uncovering of the bismuth-associated proteomes, including 63 bismuth-binding and 119 bismuth-regulated proteins from *Helicobacter pylori*, with over 60% being annotated with catalytic functions. Through bioinformatics analysis in combination with bioassays, we demonstrated that bismuth drugs disrupted multiple essential pathways in the pathogen, including ROS defence and pH buffering, by binding and functional perturbation of a number of key enzymes. Moreover, we discovered that *Hp*DnaK may serve as a new target of bismuth drugs to inhibit bacterium-host cell adhesion. The integrative approach we report, herein, provides a novel strategy to unveil the molecular mechanisms of antimicrobial metals against pathogens in general. This study sheds light on the design of new types of antimicrobial agents with multiple targets to tackle the current crisis of antimicrobial resistance.

## Abstract 2

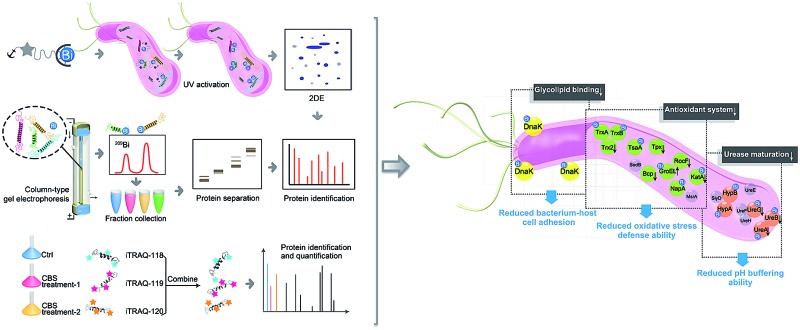
An integrative metalloproteomic approach to unveil the role of antimicrobial metals in general using bismuth as an example.

## Introduction

The rapid emergence of drug-resistant pathogens and the depletion of antibiotic pipelines pose great threats to public health.^1,2^ Alternative strategies are, therefore, urgently needed. Metal ions historically have been used as antimicrobial agents and disinfectants, in particular, certain metals such as Ag^+^ and Bi^3+^ exhibit great potential in killing multidrug-resistant bacteria^3^ and in improving the cure rates of infections from resistant strains,^4^ either when used alone or as antibiotic adjuvants. However, their modes of action are largely unexplored.^5,6^

*Helicobacter pylori* is a transmissible human pathogen that is strongly related to gastrointestinal diseases, even stomach cancer.^7^ Owing to the prevalence of antimicrobial resistance, bismuth-containing quadruple therapy has been suggested as the first-line therapy and has shown excellent success rates in the eradication of *H. pylori* and even towards established antibiotic resistant strains.^4,8,9^ Despite being used for more than 30 years in the treatment of *H. pylori* infections, bismuth-based therapies still possess efficacy towards the pathogen.^10^ Prior extensive studies show that proteins appear to be the targets of bismuth drugs.^11,12^ However, the systemic response of the pathogen to bismuth drugs has not yet been explored comprehensively, and elucidation of the secret of the sustained susceptibility of *H. pylori* to a bismuth drug could shed light on coping with antimicrobial resistance.

Although proteomics has been well adapted to track proteins regulated by metallodrugs,^13^ the systemic identification of metal or metallodrug binding proteins is particularly important towards understanding their roles in biology and medicine given that metals or metallodrugs often bind and functionally perturb the biological functions of metalloproteins and/or metalloenzymes.^11,14^ Different from proteomics, metalloproteomics focuses on the large-scale study of metals and their binding proteins, and is emerging as an invaluable tool to investigate the role of metals in cell biology and disease processes^15,16^ as well as in the elucidation of the molecular mechanisms of metallodrugs.^17^ However, it is a considerable challenge to track proteins that bind to metallodrugs, particularly in live cells, as the interaction of metals/metallodrugs with proteins *in vivo* can be weak or even transient. This makes them difficult to be identified.

**Fig. 1:**
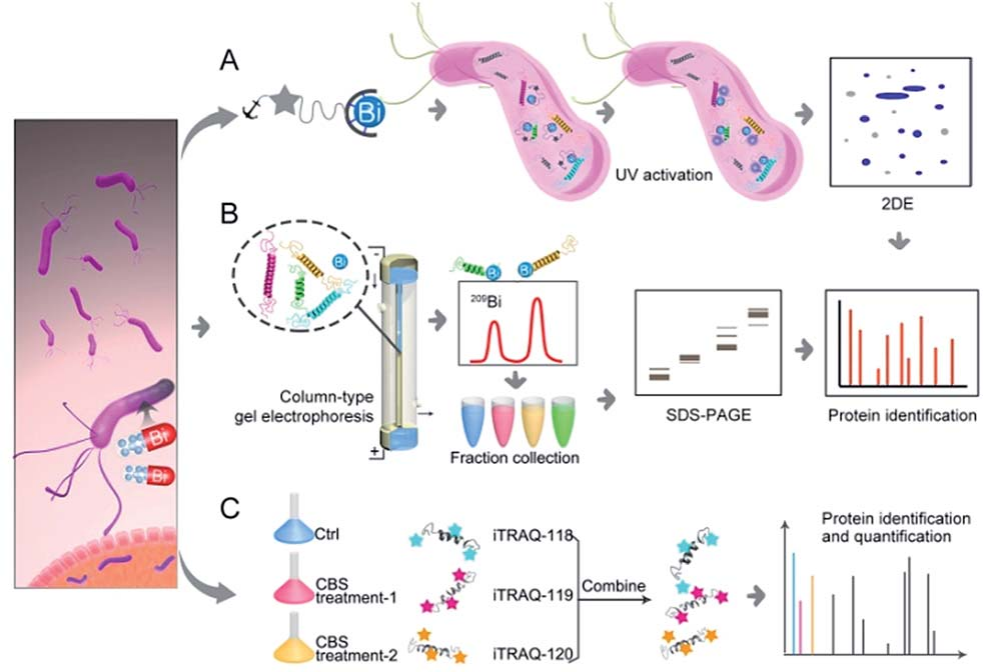
Identification of metal-associated proteomes by integration of metalloproteomics with quantitative proteomics using Bi^3+^ as an example. (A) Bi^3+^-TRACER-based approach for tracking Bi-binding proteomes in live cells, including those weakly or transiently bound. (B) GE-ICP-MS for identification of Bi-binding proteins with high affinity. (C) iTRAQ-based quantitative proteomics for profiling bismuth-regulated proteins.

Herein, we integrated in-house metalloproteomics, including a newly developed fluorescent probe-based approach (Bi^3+^-TRACER), and continuous-flow gel electrophoresis coupled with an ICP-MS (GE-ICP-MS)-based approach with quantitative proteomics (Fig. 1) to comprehensively identify bismuth-binding and bismuth-regulated proteins from *H. pylori* and identified a total of 63 Bi-binding and 119 Bi-regulated proteins in the pathogen. Subsequent bioinformatics analysis and bioassays revealed that bismuth disrupts multiple essential pathways in *H. pylori*, *e.g.* pH buffering and ROS defence. We identify and provide herein the first insight that *Hp*DnaK may serve as one of the targets of the bismuth drug to prevent host-pathogen cell adhesion. The unique multiple actions of the bismuth drug against *H. pylori* are therefore responsible for its sustained effectiveness. The integrative approach offers a novel strategy to unveil the modes of action of metallodrugs in general.

## Results

### Systemic identification of bismuth-binding and bismuth-regulated proteins in *H. pylori*

**Fig. 2:**
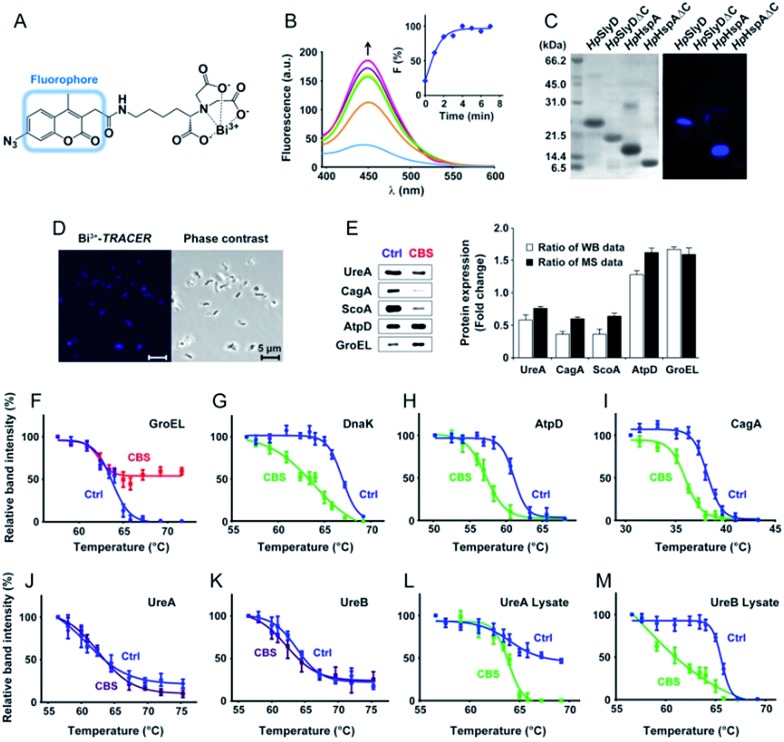
Identification and validation of the Bi-associated proteins in *H. pylori*. (A) Molecular structure of Bi^3+^-TRACER. The nitrilotriacetate (NTA) group was designed to chelate metal ions in a tetradentate manner. Upon UV activation, the arylazide moiety covalently binds to Bi-binding proteins. (B) Time-dependent fluorescence spectra of Bi^3+^-TRACER (1 μM) and normalized fluorescent intensity at *λ*_em_ = 448 nm (inset) after the addition of *Hp*HspA (10 μM). It is noted that the binding of Bi^3+^-TRACER to *Hp*HspA led to a *ca.* 5-fold fluorescence enhancement within 4 min. (C) Fluorescent labelling of His- and Cys-rich proteins by Bi^3+^-TRACER on a SDS-PAGE gel. (D) Confocal imaging of *H. pylori* cells labelled with Bi^3+^-TRACER. Bacterial cells were stained with blue fluorescence after incubation with Bi^3+^-TRACER (10 μM), indicating that the probe was diffused into *H. pylori* cells and labelled the intracellular protein targets. (E) Western blot analysis of differentially expressed proteins in CBS-treated *H. pylori*. Comparison of the altered protein expression ratios indicated a general agreement between the western blot and iTRAQ-based MS analyses. (F–M) Protein thermal melting curves of different Bi-targeting proteins in *H. pylori* intact cells (F–K) and cell lysates (L and M), treated with or without CBS. Data are presented as the mean ± SEM from at least three independent experiments.

To track bismuth-binding proteins in live *H. pylori*, we developed a bismuth fluorescent probe as a reporter, namely Bi^3+^-TRACER (Fig. 2A), by reacting equimolar amounts of Bi^3+^ (as bismuth nitrate) with a fluorescent ligand NTA-AC^18^ in a buffered aqueous solution, where the formation of Bi^3+^-TRACER was confirmed by ESI-MS (Fig. S1†). The feasibility of Bi^3+^-TRACER in labelling proteins *in vitro* was demonstrated by recombinant SlyD and HspA from *H. pylori*, which bind to Bi^3+^ *via* the unique His- and Cys-rich C-termini.^19,20^ A time-dependent increase in fluorescence was observed upon mixing *Hp*HspA with Bi^3+^-TRACER, eventually leading to a *ca.* 5-fold fluorescence enhancement (Fig. 2B). Bi^3+^-TRACER was then incubated with *Hp*SlyD, *Hp*HspA and their C-termini deleted variants (*Hp*SlyDΔC and *Hp*HspAΔC), and the samples were subjected to UV irradiation at 365 nm for 10 min to allow the formation of covalent bonds between the probe and proteins prior to SDS-PAGE analysis; intense blue fluorescence could only be observed for the wide-type *Hp*SlyD and *Hp*HspA but not for the C-termini deleted variants (Fig. 2C), confirming that Bi^3+^-TRACER could label proteins with Bi-binding abilities on a denatured gel. Upon photo-cross-linking, the dissociation of Bi did not influence the fluorescent labelling. We then incubated Bi^3+^-TRACER (50 μM) with *H. pylori* cells for 30 min and intense blue fluorescence was observed throughout the pathogen (Fig. 2D), suggesting that the probe could enter *H. pylori* cells to label bismuth-binding proteins. Similarly, the labelled proteins were anchored to the probe by photo-activation of the arylazide of the probe, and then separated by conventional two-dimensional electrophoresis (2DE) (Fig. S2†), and subsequently identified through peptide mass fingerprinting. A total of 46 bismuth-binding proteins in *H. pylori* were identified by using Bi^3+^-TRACER (Table S1†).

We then used in-house continuous-flow gel electrophoresis coupled with ICP-MS (GE-ICP-MS)^20^ to track intrinsic Bi^3+^-binding proteins (Fig. 1B). *H. pylori* 26695 cells treated with CBS (Colloidal Bismuth Subcitrate, 20 μg mL^–1^ in culture) were lysed and subsequently fractionated into soluble inner membrane (sarkosyl soluble) and outer membrane fractions (SDS soluble) using different extraction buffers. The lysates were subjected to GE-ICP-MS analysis. A total of 26 proteins, with 9 being membrane proteins (Fig. S3†), were identified by GE-ICP-MS (Table S1†), including pH-buffering enzymes, *i.e.* urease subunits (UreA and UreB), and key ROS/RNS defence enzymes, *i.e.* alkyl hydroperoxide reductase (TsaA), thioredoxin (TrxA) and catalase (KatA). These proteins, which bind strongly to bismuth even under denaturing conditions, might play crucial roles in the susceptibility of *H. pylori* towards bismuth drugs.

To profile the intact proteome of *H. pylori* in response to bismuth drugs, CBS-treated and untreated *H. pylori* 26695 protein extracts were compared in a quantitative iTRAQ experiment (Fig. 1C). A 3-Plex iTRAQ isobaric labelling strategy^21^ was applied to one control group and two CBS-treated groups. Comparative analysis of the protein expression differences between the control and two CBS-treated groups were quantified by LC-MS/MS. To improve the overall separation resolution of liquid chromatography (LC) and to increase proteome coverage, we applied an online three-dimensional (3D) RP-SCX-RP LC system by combining two reverse-phase (RP) and one strong cation exchange (SCX) mode.^22^ Using this system, 921 distinct proteins noted by at least two high-confidence peptides per protein with a confidence level of 95% and global false discovery rate (FDR) of less than 1% were identified from the *H. pylori* iTRAQ samples, and 850 of them were identified from technical duplicate runs, which achieved a high proteome coverage of *ca.* 59% (921 of 1555 predicted proteins) in *H. pylori* 26695 identified by a single technique.^23^ In total 119 proteins, including 34 up-regulated and 85 down-regulated proteins, were identified as significantly differentially expressed proteins (*p* < 0.05) with >1.30-fold or <0.77-fold change in abundance compared to the control group (Table S3 and Fig. S4†).

We then selected 6 putative CBS protein targets including GroEL, DnaK, AtpD, CagA, UreA and UreB, for verification using cellular thermal shift assay (CETSA).^24^ Supplementation of 20 μg mL^–1^ CBS to the bacterial culture medium caused the thermal melting curves of GroEL, DnaK, AtpD and CagA all to be shifted (Fig. 2F–I, Fig. S5†). However, to our surprise, we did not observe obvious thermal shifts of UreA and UreB in cells even with the supplementation of up to 200 μg mL^–1^ CBS (Fig. 2J and K), which was likely due to the fact that the low concentrations of bismuth uptaken by *H. pylori* ^25^ is unlikely to saturate both UreA and UreB given the high abundance of urease in the pathogen. Indeed, the thermal stabilities of the two proteins were shifted upon the supplementation of 200 μg mL^–1^ CBS to *H. pylori* cell lysates (Fig. 2L and M). We also verified several differentially expressed proteins identified upon bismuth treatment, including UreA, AtpD, GroEL, ScoA and CagA, by using western blotting. CBS treatment led to the expression levels of UreA, CagA and ScoA to be decreased, whereas AtpD and GroEL increased, which is consistent with iTRAQ-based quantitative analysis (Fig. 2E).

### Disruption of multiple biological pathways by bismuth in *H. pylori*

To functionally categorize the identified bismuth-associated proteins, *i.e.* bismuth-binding and bismuth-regulated proteins, we first carried out the genome-wide Gene Ontology (GO) annotation for *H. pylori* 26695 gene products as described in the supplementary method. A total of 1340 different Gene Ontology (GO) terms assigned to 1067 distinct proteins in *H. pylori* 26695 were achieved, with an annotation coverage of 69% in the total bacterium genes.

We then identified the functional PPI subnetworks that were significantly influenced by the bismuth drug in *H. pylori* using a home-made enrichment tool programmed in Python. A number of PPI subnetworks, such as tricarboxylic acid cycle (TCA, *p* = 1.05 × 10^–9^), cell redox homeostasis (*p* = 4.44 × 10^–9^), nickel homeostasis (*p* = 6.93 × 10^–9^), protein folding (*p* = 1.95 × 10^–8^) and iron homeostasis (*p* = 6.65 × 10^–8^), were identified (Fig. S6A and Table S4†), providing clues for the primary bismuth-targeting pathways in the bacterium.

We next examined the enrichment degrees of different enzymatic activities among the Bi-associated proteins (Table S5†). We narrowed our analysis down to six enzymatic activities, *i.e.* oxidoreductase activity, hydrolase activity, transferase activity, ligase activity, lyase activity and isomerase activity, as they were identified as the direct child GO terms of catalytic activity (GO:0003824) among the 1055 annotated GO terms. The enrichment analysis revealed that proteins possessing oxidoreductase activity (*p* = 5.74 × 10^–7^) were markedly influenced by CBS in *H. pylori* (Fig. S6B and C, Table S6†). Among these, AhpC is a member of the ubiquitous 2-Cys peroxiredoxins family, which h can prevent oxidative damage originating from hydrogen peroxide, peroxynitrite and a wide range of organic hydroperoxides through peroxidase activity, and is highly expressed as the most abundant antioxidant protein in *H. pylori*.^26^ Catalase is responsible for the removal of hydrogen peroxide. Thioredoxin (TrxA), GroEL, neutrophil activating protein (NapA) and the modulator of drug activity (MdaB) either facilitate the regeneration of these antioxidant enzymes or reduce indirectly the oxidative stress caused by CBS.

**Fig. 3:**
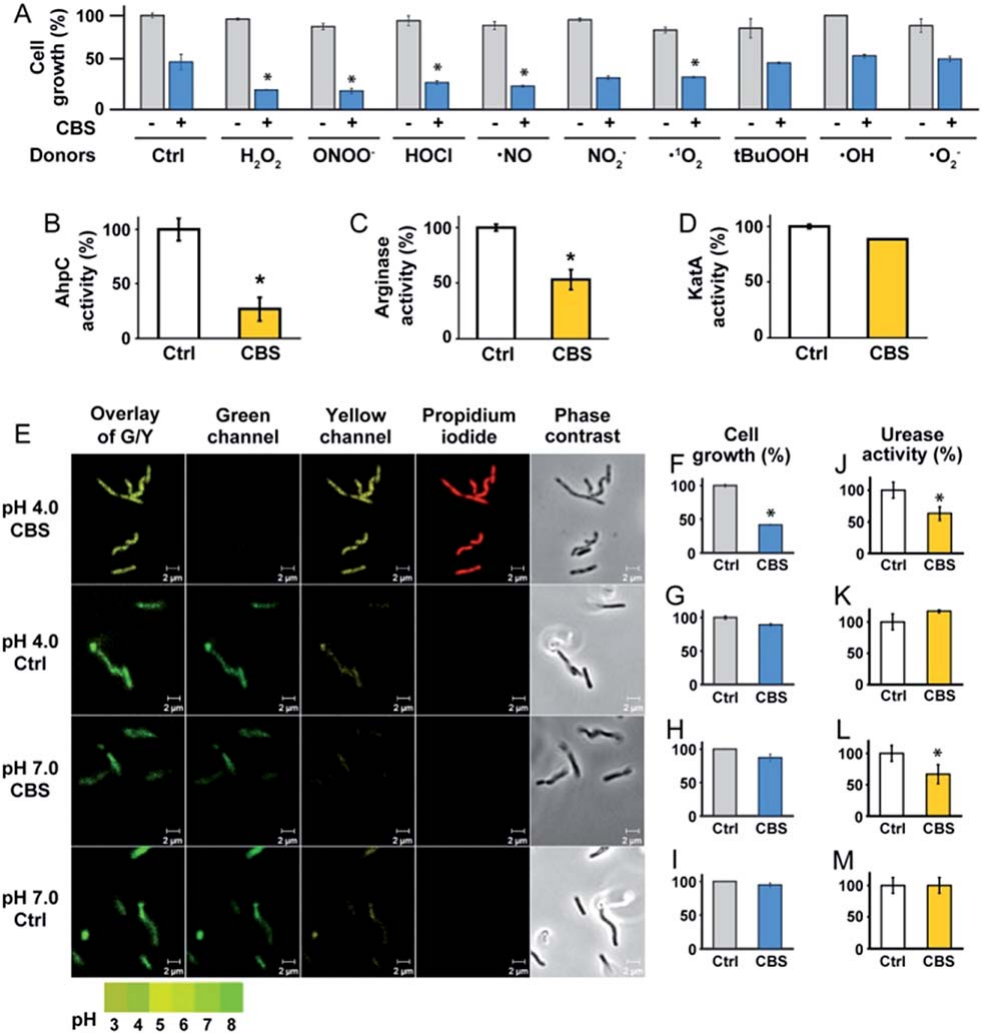
CBS inhibits the growth of *H. pylori* by attacking the oxidative stress defence and the pH-buffering systems. (A) Growth of *H. pylori* under oxidative stress from various donors without (control) and with CBS treatment. Under the stress of various ROS donors, the growth of Bi-treated bacterium was significantly lower than the control groups, implying its reduced ability for defence against these oxidative stress donors. The activities of three enzymes, AhpC, arginase and KatA, were inhibited by Bi (B–D). (E) Intracellular pH of *H. pylori* indicated by the fluorescent probe LysoSensor™. Under acidic conditions (pH = 4.0), Bi-treated bacteria exhibited acidic intracellular pH, while the untreated bacteria could keep the intracellular pH as neutral. Under neutral conditions (pH = 7.0), Bi treatment did not induce obvious changes in the bacterial intracellular pH. The growth of Bi-treated bacterium was significantly inhibited under acidic conditions (F and G) but not under neutral conditions (H and I). The differences in urease activities (J–M) implied that Bi inhibited the enzyme function. (*, *p* < 0.05).

To experimentally validate the results obtained by bioinformatics, we examined the effect of CBS on the oxidative stress defence systems in *H. pylori*. We found that bacterial growth under various oxidative stress donors was almost unperturbed in the absence of CBS (Fig. 3A), indicating that the bacterium possesses its own defence systems to cope with oxidative stress. In contrast, upon the supplementation of CBS into the cell culture medium, the growth of *H. pylori* was inhibited by nearly 70% under various oxidative stresses (Fig. 3A, Fig. S7†). We further examined the effects of CBS on several major antioxidant enzymes from *H. pylori*, *i.e.* AhpC, KatA, SOD and arginase. Upon treatment of *H. pylori* with CBS, the activities of AhpC, arginase and KatA were inhibited by 73%, 47% and 15%, respectively (Fig. 3B–D). Indeed, the binding of bismuth to the conserved Cys169 of AhpC was confirmed by IMAC, with the bismuth-binding motif ^161^HFEEHGEVCPAGW^173^ being identified.^27^ The significant inhibition of AhpC activities by CBS under the multiple turnover condition (Fig. S8†) demonstrated the interference of bismuth on AhpC regeneration. In contrast, no evident changes on the activity of SOD were observed (Fig. S9†). We then determined the ROS levels in *H. pylori* upon the treatment of CBS using a fluorescent probe HKSOX1.^28^ The green fluorescent intensities increased with the amounts of CBS (Fig. S10†), indicating the elevated levels of intracellular oxidative stress caused by CBS.

**Fig. 4:**
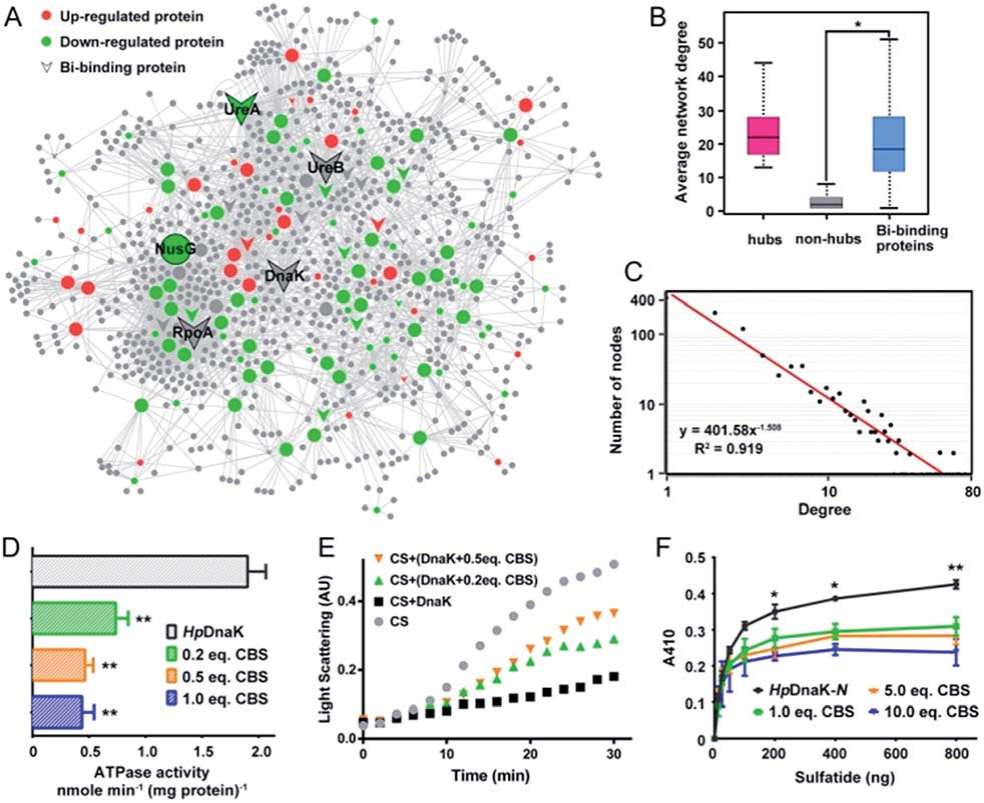
CBS targets on the hub nodes of the protein–protein interaction network in *H. pylori* and disrupts the functions of the hub protein *Hp*DnaK. (A) Bi-influenced protein–protein interaction (BiPI) network in *H. pylori*. Proteins are coloured and shaped according to their different properties in the network. Nodes in the larger size represent the central nodes with both high degree and BC values within the top 10% of the total nodes. (B) Average network degrees for hubs, non-hubs and the identified Bi-binding proteins in the BiPI network are compared. The identified Bi-binding proteins could be distinguished from the non-hub proteins in the network. (C) Degree distribution of the BiPI network in *H. pylori*. The number of nodes is plotted on the *y*-axis, and the corresponding degrees are plotted on the *x*-axis. The regression function and *R* ^2^ coefficients shown in the graph represent the power law fit yields. (D) ATPase activities of *Hp*DnaK in the absence and presence of different molar ratios of CBS (0.2-, 0.5- and 1.0-fold). (E) Influence of CBS on the chaperone activity of *Hp*DnaK examined by citrate synthase (CS) thermal aggregation assay. The light scattering of CS was monitored at 360 nm. Data show one representative result of three biological replications. (F) Glycolipid binding specificity of the N-terminal domain of *Hp*DnaK in the absence and presence of different molar ratios of CBS (1.0-, 5.0- and 10.0-fold). The asterisks indicate that the results obtained with apo-DnaK are significantly different from the Bi-bound DnaK, as compared by Student's *t* test (*, 0.01 < *p* < 0.05; **, 0.001 < *p* < 0.01).

To validate whether the bacterial pH-buffering function was influenced by CBS, we evaluated the intracellular pH of *H. pylori* grown under acidic and neutral pH conditions with or without the supplementation of CBS by a probe LysoSensor™, which gives rise to yellow or green fluorescence under acidic or neutral pH conditions, respectively. As shown in Fig. 4E and Fig. S11 (ESI†), under acidic conditions (pH = 4.0), the bacterium appeared as yellow upon CBS treatment, in contrast to green without CBS treatment, indicating the bismuth drug abolished its pH-buffering function under acidic conditions; whereas at neutral pH condition (pH = 7.0), CBS exhibited negligible influences on the intracellular pH of *H. pylori* (Fig. 3E). Consistently, upon bismuth treatment, bacterial growth was inhibited by *ca.* 70% under acidic conditions but was nearly unperturbed under neutral conditions (Fig. 3F). To further interpret this phenomenon, we examined the urease activities at different pHs with and without CBS treatment correspondingly, as the survival and colonization of the bacterium depend heavily on its ability to produce an abundance of urease. About a 40% reduction in urease activity was observed upon bismuth treatment under both acidic and neutral conditions (Fig. 3J and L).

### Bismuth targets on the key nodes of the protein interaction network in *H. pylori*

To understand the connections among the Bi-associated proteins in *H. pylori* and to explore the network architecture associated with the molecular mechanisms of action of bismuth drugs, we mapped all the identified Bi-associated proteins onto the protein–protein interaction networks in *H. pylori*. Starting from a core set of 154 Bi-associated proteins, we extracted their direct interacting proteins and constructed a Bi-influenced protein interaction (BiPI) network in *H. pylori* 26695, which resulted in one giant BiPI network composed of 967 nodes, connected *via* 2636 edges (Fig. 4A and Table S7†). Examination of the shortest paths of the network showed that two randomly selected nodes in the network were connected *via* 4.21 links, suggesting that the nodes were very closely linked.

By measuring the slope of the regression line in the cumulative distribution plot (Fig. 4C), the degree distribution of the BiPI network was fitted to a power law *P*(*x*) ∝ *x* ^–2.51^, suggesting the BiPI network followed a scale-free topology,^29^ *i.e.* a small number of highly connected proteins participating in dozens of processes and function as core proteins, while most of the other proteins have only a few connections.^29^ The nodes with large degree and high BC values were viewed as hub and bottleneck nodes in a biological network (Table S8†). Five central nodes with both high BC and large degrees within the top 1% of the total nodes were highlighted in the BiPI network (Fig. 4A). Notably, four out of the five central nodes were identified as Bi-binding proteins in *H. pylori*, including UreA and UreB, the subunits of a key enzyme urease; RpoA, the subunit alpha of DNA-directed RNA polymerase that catalyzes the transcription of DNA into RNA using ribonucleoside triphosphates as substrates; and DnaK, a major heat shock protein that could express on the bacterial surface and is involved in the modulation of glycolipid binding specificity of the bacterium.^30^ The other hub protein NusG, which is down-regulated by bismuth, participates in transcription elongation, termination and anti-termination. By examining the average degrees and betweenness values of the identified Bi-binding proteins in the BiPI network, we found that the 63 Bi-targeting proteins could not only be grouped with highly connected hub proteins, but also could be characterized as bottleneck proteins in the network (Fig. 4B and Table S9†). Given that targeting a highly connected hub protein is more likely to be lethal to an organism than targeting a non-hub protein,^31^ and it is also less likely to develop drug resistance,^32^ the unique mode of action of a bismuth drug may explain its lethality and lower likelihood to develop resistance.

To the best of our knowledge, *Hp*DnaK (Hsp70) was identified for the first time to be a potential target of bismuth drugs. We then experimentally validated this finding by overexpression and purification of the protein to examine the interaction of Bi^3+^ with *Hp*DnaK. The binding of Bi^3+^ to the cysteines of *Hp*DnaK was evidenced by the observation of a broad absorption band centred at *ca.* 360 nm in the UV-vis difference spectra (Fig. S12B†), with a stoichiometry of [Bi-NTA]/[*Hp*DnaK] of 0.5 : 1, indicating that Bi^3+^ likely coordinates to the cysteines from each *Hp*DnaK monomer. A size exclusion chromatography study showed that the binding of Bi^3+^ to *Hp*DnaK induced quaternary structural changes of the protein from a monomer to a dimer (Fig. 4D).

To examine whether the bismuth drug functionally inhibited *Hp*DnaK, we first determined the ATPase activity of *Hp*DnaK in the absence and presence of CBS. The rates of ATP hydrolysis measured by monitoring the amounts of released free phosphate were found to be linear within 80 min (Fig. S12D†). A marked decrease by 60% in the ATPase activity of *Hp*DnaK was observed in the presence of only 0.2 molar equivalent of CBS (Fig. 4D); a further decrease in the activity but to a lesser extent was seen with the addition of more CBS. We next examined the influence of CBS on the chaperone activity of *Hp*DnaK using citrate synthase (CS) as the substrate. As the heat-induced aggregation of CS was obviously influenced in the presence of CBS (Fig. S12E†), only the effects of the substoichiometric concentrations of CBS were examined, and Bi-bound *Hp*DnaK was completely desalted before the assay to ensure the absence of free bismuth. It was found that the presence of CBS disrupted the protection effect of *Hp*DnaK on the aggregation of citrate synthase to a certain extent (Fig. 4E), while the heat-induced aggregation of apo- or Bi-bound *Hp*DnaK was not observed (data not shown). We further examined the glycolipid binding property of the protein by modified ELISA. As the C terminus of *Hp*DnaK hydrophobically associates with the assay plates, which readily interferes with the results, the N terminus of *Hp*DnaK (residues 1–377), which is the major functional domain of DnaK for cell surface-associated glycolipid recognition,^33^ was expressed and purified for the assay. Dose-dependent bindings of apo- and Bi-bound *Hp*DnaK-N with sulfatide were observed at low sulfatide concentrations (<200 ng), whereas the bindings of Bi-bound *Hp*DnaK-N to sulfatide remained almost unchanged at higher sulfatide concentrations, indicative of a saturated binding. The sulfatide concentration required to reach the saturation binding was decreased by *ca.* 4-fold for Bi-bound *Hp*DnaK-N in comparison to that for apo-*Hp*DnaK-N. In the presence of 10.0 molar equivalent of CBS, additionally the binding of *Hp*DnaK-N to sulfatide was reduced by 32%, 37% and 44% at 200, 400 and 800 ng of sulfatide, respectively (Fig. 4F). Taken together, Bi-binding decreased the physiological ATPase activity, chaperone activity and glycolipid (cerebroside sulfate) binding affinity of DnaK, while glycolipid binding is a common mechanism for bacterium-host cell adhesion, indicating the potential of bismuth drugs in disruption of the interaction between *H. pylori* and its host gastric epithelial cells.

## Discussion

Proteome-wide profiling of drug targets is a common strategy to explore the mechanisms of action of many drugs and potential carcinogens.^34–36^ Unlike organic drugs, metal ions or metallodrugs often exert their roles through binding and functional perturbing of key proteins/enzymes; therefore, conventional quantitative proteomics alone is not sufficient to uncover the physiological and/or pathological biomolecular mechanisms of metals. Similar to proteomics, metalloproteomics is emerging as an important tool to reveal the roles of metals or metallodrugs in disease processes and treatment.^15–17^ Owing to the complexity of cellular metal-protein interactions, such as different binding affinity (mM to pM) and dynamics (inert/labile), the proteome-wide identification of metal-binding proteins is a significant challenge. Several metalloproteomic approaches have been established;^20,37,38^ however, each has its pros and cons. Conventional protein-based purification by liquid chromatography and metal-based detection by high-throughput tandem mass spectrometry (HT-MS/MS) and inductively coupled plasma mass spectrometry (ICP-MS) has often led to losses of metals that bind proteins weakly or transiently.^38^ Immobilized metal affinity chromatography (IMAC), albeit being frequently used to isolate and enrich metal-binding proteins and motifs, suffers from a number of drawbacks.^39^ In the present study, by integrating our in-house metalloproteomics with quantitative proteomics, we were able to uncover the maximum numbers of proteomes responsive to the drug treatment, including 63 Bi-binding and 119 Bi-regulated proteins, nearly 10-fold more than previously identified,^20,40^ thus providing a rich resource of potential drug targets in *H. pylori*.

To functionally categorize the identified bismuth-associated proteins, we first performed the genome-wide GO annotation to the *H. pylori* 26695 gene products by using an in-house enrichment tool. Notably, based on the newly constructed *H. pylori* GO database, we found that over 60% of the identified Bi-associated proteins were annotated with catalytic functions, which is in accordance with the hypothesis that antimicrobial metal species mainly abolish the activities of various enzymes to exert their toxicities in bacteria.^5^ Bioinformatics analysis showed that bismuth disrupts multiple biological pathways critical for the pathogen. Among the major enzymatic activities in *H. pylori*, oxidoreductase and hydrolase are the two major enzymatic activities that are significantly influenced by the bismuth drug. Defence against oxidative stress is crucial for *H. pylori* survival as the bacterial infection could induce inflammatory response, resulting in an oxidative burst, which would accumulate in the stomach and rapidly eliminate the bacterium in the absence of appropriate defences.^41^ Similarly, the survival and colonization of the bacterium depend heavily on its ability to produce an abundance of urease, a cytosolic metalloenzyme that catalyzes the hydrolysis of urea to ammonia, which effectively buffers its intracellular pH and creates a neutral layer around the bacterium,^42,43^ which is important given the special niches in which *H. pylori* resides. Subsequent experimental validation showed that bismuth drugs impair the oxidative defence systems and abolish the bacterial pH-buffering ability, possibly by binding and functionally disrupting key enzymes.

Furthermore, based on the Bi-influenced protein interaction network in *H. pylori*, we found that the identified Bi-binding proteins had a high tendency to be grouped with hub proteins in the network. Given that highly connected hub nodes are generally essential for the integrity and stability of a PPI network, disruption of the function of a highly connected hub protein is more likely to be lethal to the organism than targeting a non-hub protein.^44–46^ Moreover, targeting hub proteins by a drug is also featured as less likely to develop drug resistance, as proteins with more interactors are more tolerant to mutations.^47^ Five central nodes were uncovered in the BiPI network, with four of them being Bi-binding proteins in *H. pylori*. Apart from UreA and UreB, subunits of urease, which is well recognized to be one of the targets of bismuth drugs,^20,40^ DnaK, RpoA and NusG were also situated in the central nodes, and appeared to be newly identified targets of bismuth drugs. We selected DnaK for further validation by purification of the protein and *in vitro* examination of the binding and functional inhibition by CBS. A biophysical study and enzymatic assay showed that Bi^3+^ indeed bound to DnaK and decreased the physiological ATPase activity and glycolipid (cerebroside sulfate) binding affinity of DnaK. While the latter is a common mechanism for bacterium-host cell adhesion,^48,49^ this provides the first molecular insight into bismuth inhibition of the interaction between *H. pylori* and its host gastric epithelial cells.^50^

## Conclusions

**Fig. 5:**
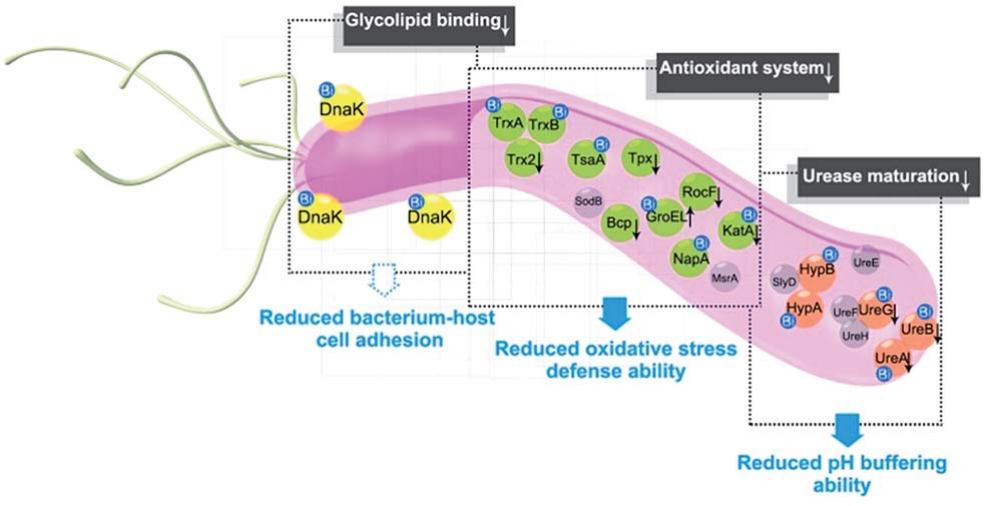
A model for the multi-targeted mode of action of CBS in eradicating *H. pylori*. As experimentally validated in the current study, bismuth drugs disrupt the oxidative stress defence and pH-buffering abilities in *H. pylori*, and inhibit the normal functions of a newly identified potential key target *Hp*DnaK, leading to various deleterious effects on the bacterium.

In summary, the integration of metalloproteomics with quantitative proteomics, in combination with bioinformatics analysis and bioassays, enabled the systemic characterization of the cellular response triggered by metal-based therapies. Our studies demonstrate that the multi-targeted mode of action of a bismuth drug (Fig. 5, Fig. S13†) is accountable for its sustainable antimicrobial activity against *H. pylori* and the low likelihood of *H. pylori* developing resistance to bismuth drugs. This study may shed light on the design of new types of antibiotics or the reuse of metals to enhance the therapeutic effects of conventional antibiotics to relieve the current crisis of antimicrobial resistance. The integrative approach we report herein provides a general platform for understanding the physiological and pathological roles of metals as well as the mechanism of metallodrugs.

## Author contributions

H. S., Y. W., H. L., I. K. C. and Z. C. conceived the project. H. S., H. L., L. H. and Y. W. designed the experiments and wrote the manuscript. Y. W., L. H., Q. Q., Y. T. L., Y. Y., Y. Y. C. and X. Y. performed the experiments. F. X., J. W. performed the bioinformatics.
