# Supplementary material for: Integrative approach for the analysis of the proteome-wide response to bismuth drugs in Helicobacter pylori
Source: Chem Sci. 2017 Apr 19;8(6):4626–33. doi: 10.1039/c7sc00766c (PMC5471454; doi:10.1039/c7sc00766c)
Supplement: Supplementary file 1 [file SC-006-C7SC00766C-s001.pdf]

Electronic Supplementary Material (ESI) for Chemical Science.
This journal is © The Royal Society of Chemistry 2017

Electronic Supplementary Information

Integrative approach for analysis of proteome-wide response to bismuth drugs in *Helicobacter pylori*

Yuchuan Wang,‡[ab] Ligang Hu,‡[a] Feng Xu,‡[c] Quan Quan,[a] Yau-Tsz Lai,[a] Wei Xia,[b] Ya Yang,[a] Yuen-Yan Chang,[a] Xinming Yang,[a] Zhifang Chai,[d] Junwen Wang,[cef] Ivan K Chu,[a] Hongyan Li[a] and Hongzhe Sun*[ab]

[a.] Department of Chemistry, The University of Hong Kong, Hong Kong, P.R. China;

[b.] School of Chemistry, Sun Yat-sen University, Guangzhou, P.R. China;

[c.] Center for Genome Sciences, The University of Hong Kong, Hong Kong, P.R. China;

[d.] CAS Key Laboratory of Nuclear Analytical Techniques, Institute of High Energy Physics, Chinese Academy of Sciences, Beijing, P.R. China

[e.] Center for Individualized Medicine, Department of Health Sciences Research, Mayo Clinic, Scottsdale, AZ 85259, USA;

[f.] Department of Biomedical Informatics, Arizona State University, Scottsdale, AZ 85259, USA.

‡These authors contributed equally to this work.

*Correspondence and request materials should be addressed to H.S. (e-mail: hsun@hku.hk).

Supplementary Methods and Figures

Culture of *H. pylori*. *Helicobacter pylori* 26695 (American type culture collection, ATCC 700392) glycerol stock was recovered from -80 ℃ and cultured on columbia agar plates supplemented with 7% laked horse blood (Oxoid) and selective antibiotics (Oxoid). The agar plates were incubated at 37 ℃ for 3 days under microaerobic conditions (5% CO_2, 4% O_2 and 91% N_2) generated by CampyGen (Oxoid). Cells were then transferred into Brucella broth (BD) supplemented with 0.2% β-cyclodextrin (Sigma) and cultured with 105 rpm agitation at 37 ℃. *H. pylori* cells at early log phase (OD_{600} = 0.1-0.2) were subjected to CBS (colloidal bismuth subcitrate, Livzon Pharmaceutical Ltd) treatment. Liquid cultures after *ca*. 24 h incubation were harvested for further experiments.

Identification of Bi-binding proteomes in *H. pylori*. Two different approaches were applied to the proteome-wide identification of Bi-binding proteins in *H. pylori*. To label Bi-binding proteins with the fluorescent probe Bi^{3+}-*TRACER*, *H. pylori* liquid culture grown to mid-log phase (OD_{600} = 0.5) were incubated with 50 μM of Bi^{3+}-*TRACER* at 37 ℃ in dark for 30 min. Cells were then exposed under ultraviolet radiation at 365 nm for 15-20 min, and centrifuged to discard the supernatant. An aliquot of *H. pylori* cells resuspended in PBS buffer (OD_{600} = 0.3) was subjected to confocal imaging. Images were captured by a Carl Zeiss LSM700 Inverted Confocal Microscope, with a Plan-Apochromat 63×/1.40NA/oil-immersion objective for fluorescent and phase contrast imaging.

H. pylori soluble and membrane proteins were extracted by lysing the cell pellets resuspended in Tris buffer A (20 mM Tris, 100 mM NaCl, 1 mM TCEP, 0.5 mM PMSF, pH 7.2). After discarding the unbroken cells centrifuged at 2,000 *g*, the remaining suspension was subjected to ultracentrifuge at 48,000 *g* for 30 min at 4 ℃, and the supernatant containing cytosolic and periplasmic proteins were collected. The cell debris were further washed and dissolved in Tris buffer A supplemented with 1% SDS to obtain the membrane proteins. Protein concentrations were determined by BCA assay (PIERCE), and the soluble proteins were subjected to 2DE separations. For 2DE analysis, isoelectric focusing (IEF) was performed using a precast Immobiline DryStrip (13-cm, pH 4-7 NL, GE Healthcare), followed by the second dimensional SDS-PAGE (13.5% acrylamide gel) separation. Fluorescent spots on gels were imaged by ImageQuant 350 (GE Healthcare) after electrophoresis, and then subjected to silver staining for protein visualization. Protein spots corresponding to the fluorescent spots were excised from the gels, subjected to trypsin digestion and desalting treatment. The molecular masses of the digested peptides were

determined by MALDI-TOF/TOF-MS analysis and the data were searched against NCBInr protein database using the MASCOT searching engine.

As complementary analysis, CBS-treated (20 μg/mL) *H. pylori* protein fractions were further subjected to GE-ICP-MS analysis. As Bi-binding proteins in *H. pylori* cytosolic fractions had been examined by GE-ICP-MS in a previous report,[1] mainly membrane fractions were analyzed herein. Briefly, appropriate amount of protein samples mixed with 2 × SDS loading buffer were loaded onto a freshly prepared column gel, which was composed of multilayered SDS-polyacrylamide gels. For protein identification, a T-connection tubing was employed to split the solution after electrophoresis, and protein fractions corresponding to the major metal peaks were collected and subjected to SDS-PAGE separation and protein identification.

***H. pylori* protein extraction, digestion and iTRAQ labeling.** For quantitative evaluation of the changes in *H. pylori* proteome upon drug treatment, three groups (one control and two CBS-treated groups, 20 μg/mL) of *H. pylori* liquid cultures were harvested and lysed in Hepes buffer (20 mM Hepes, 500 mM NaCl, 1mM TCEP, 0.1% Triton X-100, pH 7.2) for iTRAQ labeling. On-filter protein digestion was performed as previously described[2] with some modifications. 100 μg proteins of each treatment was denatured by 10 mM dithiothreitol (DTT) at 60 ℃ for 30 min, followed by the incubation with 20 mM iodoacetmide (IAA) in 100 mM ammonium bicarbonate buffer in dark for 1 h (for cysteine-blocking), and then centrifuged at 3,000 *g* to load on Microcon® centrifugal filters for protein purification. A volume of 100 μL milliQ H_2O was added on the filter and removed by centrifugation at 3,000 *g* to clean up possible remaining chemicals on the filter other than proteins. Samples were then proteolyzed with 1:33 sequencing-grade trypsin in 0.5 mM triethylammonium bicarbonate (TEAB) buffer at 37 ℃ overnight. All three trypsinized samples were subsequently collected by centrifuging at 3,000 *g*, and labeled with the corresponding iTRAQ labeling reagent at room temperature for 2 h, following the manufacturer’s instructions (iTRAQ reagent 118: control; 119: CBS-1; 120: CBS-2). The iTRAQ samples were dried by SpeedVac before storing at -80 ℃ till use.

iTRAQ-labeled protein analysis and data processing. The iTRAQ-labeled protein samples were reconstituted in water. For each technical replicated run, 30 μg of the digested proteins were analyzed using a three-dimensional RP-SCX-RP separation system constituted with an Eksigent nanoLC Ultra 2D Plus system (AB SCIEX, Framingham, MA) slightly modified by adding one extra strong anion exchange (SAX) dimension for separation.[3] Twelve fractions

were applied for the first high-pH RP dimension, each divided into one SCX and one SAX sub-fractions. Low-pH final RP dimension provided an organic gradient from 5% to 35% ACN in H_2O within 90 min.

The MS data were acquired on a TripleTOF 5600 system (AB SCIEX, Framingham, MA). The MS parameters used were as follows: ion spray voltage, 2.8 kV; curtain gas, 20 psi; nebulizer gas, 6 psi; interface heater temperature, 125 ℃. For information-dependent acquisition (IDA), full scans were acquired within 250 ms over the range *m/z* 400-1250, followed by MS/MS scans of the 20 most abundant peaks that exceeded 125 counts per second and carried a charge between +2 to +5 in the range *m/z* 100-1500. The dynamic exclusion time of the acquired ions was set at 20 s. The acquired MS/MS data were analyzed using the Paragon algorithm in ProteinPilot 4.0 software (Applied Biosystems, Framingham, MS, USA), and were searched against *Helicobacter pylori* (strain 26695) in Uniprot database. TripleTOF 5600 data files were converted to .mgf files using default parameters of PeakView 1.1 before being searched using ProteinPilot 4.0 version in the database. The basic local alignment search tool (BLAST) was used to search sequence similarities of hypothetical or uncharacterized proteins.

Cellular thermal shift assay (CETSA). For CETSA experiments,[4] control and CBS-treated *H. pylori* cells were harvested and washed with chilled PBS for three times. Equal amounts of cell suspensions (*c.a.* 10^9 cells) were aliquoted into PCR tubes and heated at different temperatures for 3 min, followed by cooling for 3 min at room temperature, and the heat-cool cycles were repeated three times. The cells were then lysed by 3 freeze-thaw cycles using liquid nitrogen, and were subsequently centrifuged at 20,000 *g* for 20 min at 4 ℃ to pellet cell debris and the precipitated proteins. The supernatants were separated by SDS-PAGE followed by western blot analysis.

For the cell lysate CETSA experiments, *H. pylori* cells at mid-log phase were harvested and lysed in Hepes buffer. The cell lysates were separated from the cell debris by centrifugation, and incubated (500 μg/mL cell lysates) with different concentrations of CBS ranging from 20 to 500 μg/mL at room temperature for 30 min. Equal amounts of CBS-treated and untreated cell lysates were aliquoted into PCR tubes and individually heated at different temperatures for 3 min, followed by cooling for 3 min at room temperature for three cycles. Soluble proteins were separated by centrifugation at 20,000 *g* for 20 min at 4 ℃, and subjected to SDS-PAGE and western blot analysis. All the thermal melting curves of proteins

in intact cells and cell lysates were represented as mean ± S.D. of at least three independent experiments.

Western blot analysis. After SDS-PAGE separation, proteins of interest were transferred to PVDF membranes using 90 V constant voltage for 1.5 h. Membranes were blocked with 5% (w/v) BSA in TBST buffer for 2 h at room temperature, and subsequently incubated with primary antibodies overnight at 4 ℃ with optimized dilution ratios. After being washed with TBST buffer for three times, the membranes were further incubated with diluted secondary antibodies for 2 h at room temperature, and incubated in chemiluminescent substrate working solution (Thermo) after washing for the detection of HRP on the membranes.The antibodies used were rabbit anti-urease α (Santa Cruz), mouse anti-CagA (Santa Cruz), mouse anti-AtpB antibody (Abcam), rabbit anti-*Helicobacter pylori* urease B antibody (Abcam), rabbit anti-HSP60 antibody (Proteintech), rabbit anti-HSP70 antibody (Proteintech), rabbit anti-OXCT1 antibody (Proteintech), anti-mouse IgG-HRP (Promega) and goat anti-rabbit IgG-HRP (Bio-Rad). Western blot results were imaged with GeneGnome XRQ system (Syngene). Protein band densities were analyzed using ImageJ software. The total protein densities in each lane of the SDS-PAGE gel were quantified as loading control for protein normalization.

Bioinformatics. The genome-wide Gene Ontology (GO) annotation to *H. pylori* 26695 gene products was obtained by combining the annotations from different sources, i.e., Uniprot database, Blast searching and Ortholog detection using *Pseudomonas aeruginosa* (strain PA7) and *Escherichia coli* (strain ATCC 8739 / DSM 1576 / Crooks) as model organisms. According to the *through-path* rule, the annotation of a protein inherited all its ancestor GO terms based on the GO hierarchical ontology. Based on the three independent methods, 642, 1055 and 468 GO terms were annotated to 1004, 1064 and 1067 proteins in *H. pylori*, respectively. The complete annotation results can be viewed in the supplementary materials.

Enrichment analysis was performed between the lists of Bi-associated proteins and the PPI subnetworks in *H. pylori* to identify statistically enriched functional subnetworks modulated by Bi using an in-house enrichment tool programmed in Python. 438 GO terms annotating to at least four different proteins were considered in the enrichment analysis. The enrichment *p*-values were calculated on the basis of hypergeometric distribution, using the protein number in *H. pylori* 26695 as the background list. The Benjamini-Hochberg method was applied to adjust *p*-values for false discovery rate (FDR) control. The enriched GO terms were selected with a significance threshold of FDR < 0.05.

The complete protein-protein interaction (PPI) networks in *H. pylori* (as HPI network) were constructed by combining the high confidence PPIs (combined score > 700) of *H. pylori* 26695 from String database v9.1, the yeast two-hybrid screened high quality PPIs (prey count < 15)[5, 6] and a set of literature-curated binary protein interactions in *H. pylori*. The identified Bi-influenced proteins in *H. pylori* were mapped to the HPI network to extract their direct interacting proteins. The PPI networks and the concept gene network were visualized with Cytoscape v2.8.3. Each node was labeled by gene name or by GO term name.

Evaluation of intracellular pH of *H. pylori*. Intracellular pH of *H. pylori* was evaluated by incubating the CBS-treated/untreated bacteria with a pH-dependent fluorescent probe LysoSensor™ Yellow/Blue (1 μM, Invitrogen L7545) in acidic (pH 4.0) or neutral (pH 7.2) PBS buffer (supplemented with 5 mM urea and 25 μM Ni^{2+}) under microaerobic conditions at 37 ℃ for 30 min. Cells were then centrifuged and resuspended in the corresponding PBS buffer with different pH values. An aliquot of bacteria with OD_{600} = 0.3 was added to the confocal dish and subjected to confocal imaging. The fluorescence signals at 440and 540 nm were excited using a 405 nm laser source. Intracellular pH was evaluated based on the ratio of fluorescence intensity at 540/440 nm. The viability of *H. pylori* was indicated by the fluorescence intensity of propidium iodide (1 μg/mL) measured at 630 nm upon excitation using a 555 nm laser source.

Evaluation of urease activity of *H. pylori*. Urease activity of *H. pylori* was evaluated by pheno-hypochlorite assay as described previously.[7] CBS-treated/untreated *H. pylori* cells incubated under different pH conditions (in PBS buffer, under microaerobic conditions for 30 min) were harvested and lysed in Hepes buffer. For a volume of 250 μL supernatant, 375 μL solution A (10 g/L phenol and 50 mg/L sodium nitroprusside) was added, followed by the addition of 375 μL solution B (0.044% sodium hypochlorite in 5 g/L sodium chloride solution). Reactions were conducted at 37 ℃ for 30 min and the absorbance was measured at 620 nm. The amounts of released ammonia were calibrated using a series of ammonium chloride standard solutions. One unit of urease activity was defined as the amount of urease required to catalyze the production of 1 μmol ammonia per min per mg of total proteins.

The effects of reactive oxygen/nitrogen species on the growth of *H. pylori*. The dose-response relationship of major oxygen/nitrogen species (ROS/RNS) against the growth of *H. pylori* was first determined. The stress species H_2O_2, HOCl, ·NO_2 and *t*BuOOH were directly prepared from the stock of hydrogen peroxide (Sigma), sodium hypochlorite (Acros), sodium nitrite (Sigma) and *tert*-butyl hydroperoxide (Sigma-Aldrich). Other stress species such as

superoxide anion ($\cdot O_2^-$) was generated from the xanthine/xanthine oxidase (Sigma) system; hydroxyl radical ($\cdot OH$) was generated from Fenton reaction by mixing Fe^{2+} (as $FeSO_4 \cdot 7H_2O$, Sigma) with H_2O_2; singlet oxygen ($^1O_2$) was generated by mixing MoO_4^{2-} (as $Na_2MoO_4 \cdot 7H_2O$, Merck) with H_2O_2; nitric oxide ($\cdot NO$) was generated from NOC-18 ([2,2'-(Hydroxynitrosohydrazino)bis-ethanamine], Merck); and peroxynitrite ($ONOO^-$) was generated from SIN-1 ([3-(4-morpholinyl)sydnone imine hydrochloride], Invitrogen).[8] Different concentrations of the various ROS/RNS donors were mixed with *H. pylori* cells in PBS buffer with OD_{600} = 1.0, and incubated under microaerobic conditions at 37 °C for 60 min. Cells were then harvested and inoculated to 1 mL culture medium with initial OD_{600} = 0.4, and incubated under microaerobic conditions for 72 h. The OD_{600} values were used to quantify cell growth. The normalized final/initial OD_{600} values corresponding to different ROS/RNS groups were fitted to the dose-response model respectively, and the EC_{10} values indicating the concentrations of ROS/RNS donors leading to 10% effective response against the growth of the pathogen were determined accordingly.

The EC_{10} and 1/6 EC_{10} values of ROS/RNS donors were used for evaluation of the ability of *H. pylori* to defense against oxidative stress. Experimental procedures were similar to that of determining the dose-response relationship. The desired concentrations of the various donors were added to the culture medium with or without the addition of CBS (20 μg/mL).

Evaluation of the activities of key redox enzymes in *H. pylori*. CBS-treated/untreated *H. pylori* cells (grown to mid-log phase, 20 mL, 20 μg/mL CBS) lysed in 5 mL Hepes buffer (10 mM Hepes, 100 mM NaCl, 0.5 mM TCEP, pH 7.4) were subjected to enzymatic activity tests.

The enzymatic activity of AhpC was evaluated *viatert*-butyl hydroperoxide (*t*BHP) consumption with FOX assay.[9] Briefly, 1 mL cell lysates mixed with 100 μM DTT (without DTT in single turnover test) and 100 μM *t*BHP were incubated at 37 °C for 30 min. The reaction was quenched by removing 20 μL solution from the assay and adding to 200 μL of FOX working reagent, which was prepared by pre-mixing FOX reagent A (25 mM ferrous ammonium sulfate in 2.5 M sulfuric acid) with FOX reagent B (100 mM sorbitol and 125 μM xylenol orange in water) in a 1:100 volume ratio. Samples were mixed and incubated at room temperature for 30 min for stabilization, and the absorbance was measured at 560 nm. The peroxide concentration was calculated from a series of *t*BHP standard solutions.

The enzymatic activity of catalase was evaluated *via* H_2O_2 consumption with FOX assay.[10] Briefly, 1 mL cell lysates mixed with 200 μM H_2O_2 were incubated at room temperature for 15 min. The reaction was quenched by removing 10 μL solution from the

assay and adding to 190 μL of FOX1 reagent (250 μM ammonium ferrous sulfate, 100 μM xylenol orange, 0.1 M sorbitol and 25 mM H_2SO_4). Samples were mixed and incubated at room temperature for 30 min, and the absorbance was measured at 560 nm. The residual H_2O_2 was calculated from a series of H_2O_2 standard solutions.

Superoxide dismutase (SOD) activity was monitored by measuring the inhibitory effect of the enzyme on the reduction of ferricytochrome *c via* the competition for superoxide generated from xanthine.[11] In brief, appropriate amount of xanthine oxidase solution was added to 1 mL SOD assay cocktail (50 mM PBS, 0.1 mM EDTA, 50 μM xanthine and 10 μM cytochrome *c*), to obtain a rate of increase in absorbance at 550 nm in the range of 0.015 to 0.025 A/min. Subsequently, *ca.* 40 μL cell lysates were mixed with 960 μL SOD assay cocktail and the same amount of xanthine oxidase solution as determined in the previous step. The inhibitory effect of SOD was reflected at the lower rate of increase in absorbance at 550 nm under the assay conditions, and the % inhibition was controlled in the range of 40% to 60% to obtain more accurate calculations. One unit of activity is the amount of SOD required to inhibit the reduction of ferricytochrome *c* by 50%.

Arginase activity was measured by monitoring the conversion rate of L-arginine to ornithine.[12] In brief, 500 μL cell lysates were pre-incubated with 500 μL 10 mM cobaltous chloride at 55 ℃ for 30 min to activate the enzyme. 50 μL arginase buffer (20 mM Tris-HCl, 10 mM L-arginase, pH 7.5) was subsequently added and the incubation was continued at 37 ℃ for 1 h. The reaction was quenched by removing 10, 50, 100 μL solution from the assay and adding to 750 μL acetic acid, respectively, followed by the incubation with 250 μL ninhydrin (4 mg/mL) at 95 ℃ for 1 h. The absorbance was measured at 515 nm and the production of ornithine was calculated from a series of standard ornithine solutions.

Expression and purification of *H. pylori* DnaK (Hsp70). The *dnaK* gene was amplified by PCR from *H. pylori* 26695 genomic DNA with primer pairs DnaK(*Bam*HI)-for (5'-TAATGGATCCATGGGAAAAGTTATTGGAATTGATTTA-3') and DnaK(*Eco*RI)-rev (5'-ATTAGAATTCTCACTCCACTTCCGCATCAAT-3') (the restriction sites are underlined). The digested DNA fragments were purified and ligated into the same digestion sites of pGEX-4T-1 vector. The *dnaK-N* gene was amplified with the primer pairs DnaK-*N*(*Bam*HI)-for (5'-TAATGGATCCGATGGGAAAAGTTATTGGAATTGATTTA-3') and DnaK-*N*(*Eco*RI)-rev (5'-ATTAGAATTCTCATTTCAACACGCCCCCTTG-3'), and ligated into pET-47b (+) vector. The constructed plasmids were sequenced to confirm that no mutation occurred during the manipulation.

Escherichia coli BL21 (DE3) cells harboring the expression vector were grown in LB medium supplemented with antibiotics at 37 ℃ to an OD_{600} of 0.6-0.8, followed by the addition of 0.2 mM isopropyl β-D-thiogalactoside (IPTG) to induce protein expression. After further culture for 16 hrs at 25 ℃, the cells were harvested by centrifugation (5,000 *g*, 20 min) and resuspended in Tris buffer B (20 mM Tris, 300 mM NaCl, pH 7.2). The cells were lysed by homogenization, and centrifuged (20,000 *g*, 30 min) to remove cell debris.

Supernatant of *E. coli* cells overexpressing GST-fused *Hp*DnaK was subjected to GST Sefinose^{TM} Resin (Sangon). After washing thoroughly with GST binding buffer (50 mM Tris, 150 mM NaCl, pH 7.5), the bound proteins were eluted by the same buffer in the presence of 20 mM reduced glutathione (Sigma-Aldrich), followed by thrombin (50 NIH units) cleavage at 22 ℃ for 16 hrs to remove the GST tag. After further incubation with GST resin, the eluted fractions were concentrated and purified by gel filtration using a Hiload 16/60 Superdex 75 column (GE Healthcare).

For purification of recombinant protein *Hp*DnaK-*N* (residues 1-377), *E. coli* cell lysates were subjected to HisPur^{TM} Ni-NTA Resin equilibrated with Tris buffer B supplemented with 5 mM imidazole. The bound proteins were eluted by 300 mM imidazole-containing Tris buffer B and desalted to remove imidazole, followed by Prescission Protease cleavage at 4 ℃ for 4 hrs. The purified *Hp*DnaK-*N* protein was obtained after further incubatingthe digested protein mixture with Ni-NTA resin to remove the un-cleaved His-DnaK-*N* and the His-tag.

Functional evaluation of the inhibitory effects of bismuth on *Hp*DnaK. The ATPase activity of *Hp*DnaK was determined by using the Malachite Green phosphate assay kit (Cayman). The reaction mixture consisting of 200 μM ATP, 1 mM $MgSO_4$, 2 μM protein (apo- and Bi-bound *Hp*DnaK) in the reaction buffer (20 mM Hepes, 100 mM NaCl, 1% glycerol, pH 7.5) was incubated at 37 ℃. Aliquots (50 μL) of the reaction mixture was taken out in a time course for determination of free phosphate. The amount of phosphate released in the reaction was obtained by subtraction of the self-hydrolysis of ATP.

The chaperone activity of *Hp*DnaK was performed using citrate synthase (CS) as substrate protein.[13] Light scattering of CS upon thermal aggregation was monitored in a Circular Dichroism (CD) spectrophotometer (JASCO J-810) in stirred and thermostatted quartz cells. During the measurement, both excitation and emission wavelengths were set to 360 nm with voltage setting to 800-850 V and slit setting to 3-4 mm. Thermal aggregation of 0.15 μM citrate synthase (Sigma) in the absence or presence of 5 molar equivalents of apo- or Bi-bound *Hp*DnaK in 50 mM Tris buffer (pH 8.0) was monitored at 43 ℃ for 30 min. To

obtain Bi-bound *Hp*DnaK, freshly prepared *Hp*DnaK was incubated with substoichiometric concentrations of CBS overnight at 4 ℃ and desalted by HiTrap desalting column (5 mL, GE Healthcare) before the assay to make sure the complete removal of the unbound bismuth.

Interaction of *Hp*DnaK-*N* with glycolipids was assessed as previously described with minor modifications.[14] Stock solution of cerebroside sulfate was prepared in ethanol with a final concentration of 40 ng/μL. 96-well plates were inoculated with different amounts of lipids (0-800 ng/50 μL/well) and dried overnight at room temperature. The wells were blocked with 250 μL/well blocking buffer (5% BSA in 50 mM TBS, pH 7.4) at 37 ℃ for 1 h. All the following steps were performed at room temperature. After removing the blocking buffer, apo- and Bi-bound *Hp*DnaK-*N* were added to the wells and incubated for 2 hrs. Following washing with Buffer C (0.2% BSA in 50 mM TBS, pH 7.4) for three times, anti-Hsp70 antibody (1:1000 dilution in Buffer C) was added in 100 μL/well and incubated for 1 h. The plates were washed with Buffer C, and incubated with 100 μL/well goat anti-rabbit IgG-HRP antibody (1:3000 dilution in Buffer C) for 1 h. After a final wash, ABTS (2,2'-Azino-bis [3-ethylbenzothiazoline-6-sulfonic acid]-diammonium salt) substrate solution (Thermo) was added to the wells in 100 μL aliquots. The plates were covered with tin foil and allowed to develop in the dark for 20-30 min, and the reaction was stopped with the addition of 100 μL 10% SDS. Protein-glycolipid binding was determined by measuring the absorbance of each well at 410 nm. Plotted values represent the mean of triplicates subtracted the plate background values, which were taken as the absorbance readings of protein bound to glycolipid-free wells.

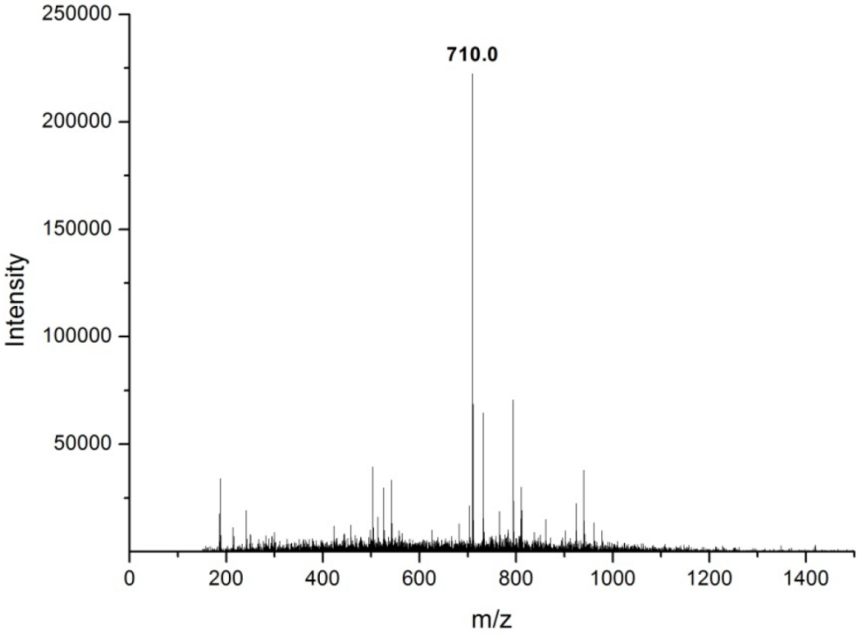

Fig. S1 ESI-MS spectra showing 1:1 complexation of *TRACER* with Bi^{3+}. The peak at 710.0 is corresponding to $[M\text{-}3H]^-$ (calcd. 710.0).

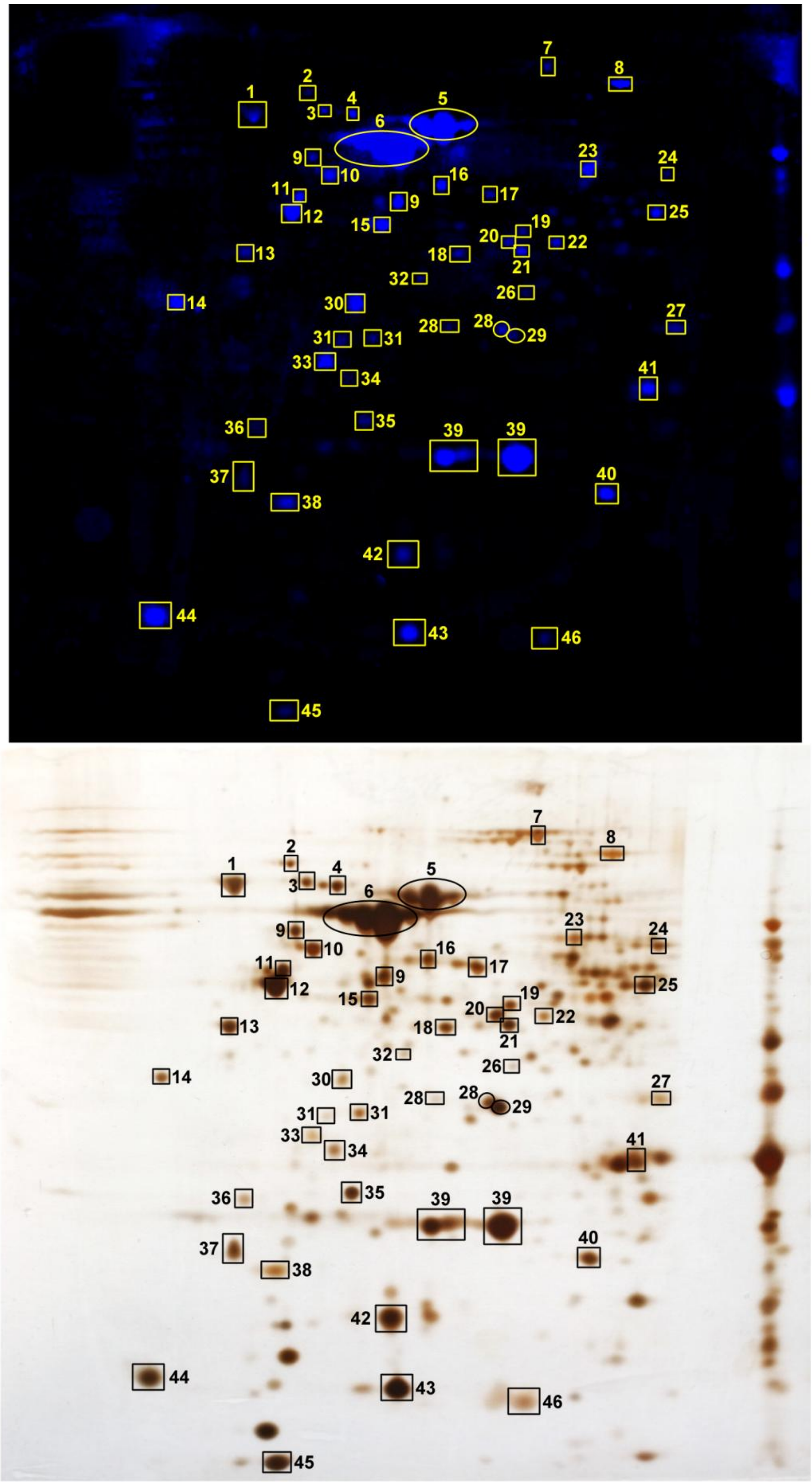

Fig. S2 2-DE gel of *H. pylori* 26695 cell lysates pre-incubated with Bi^{3+}-*TRACER*. The gel was imaged under UV exposure at 365 nm (*upper*) and then silver stained (*lower*). The boxed protein spots were excised and subjected to peptide mass fingerprinting for protein identification.

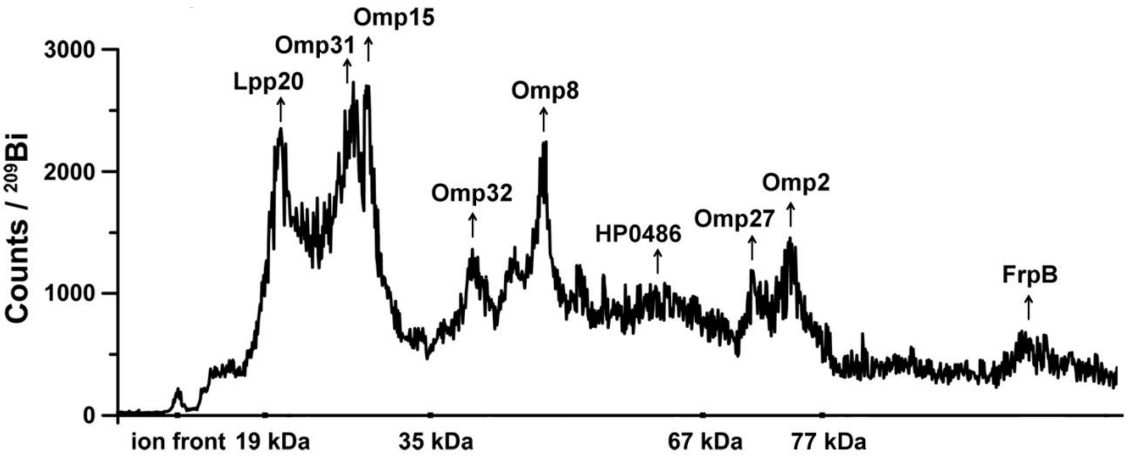

Fig. S3 Profile of Bi-binding proteins in the membrane fraction of *H. pylori* 26695 analyzed by GE-ICP-MS. The *x*-axis is plotted according to the molecular weights of a series of iodine-labeled standard proteins. Bi-associated peaks are mainly observed in the molecular weight range of less than 77 kDa. The identified proteins corresponding to the major ^{209}Bi peaks are indicated on the graph.

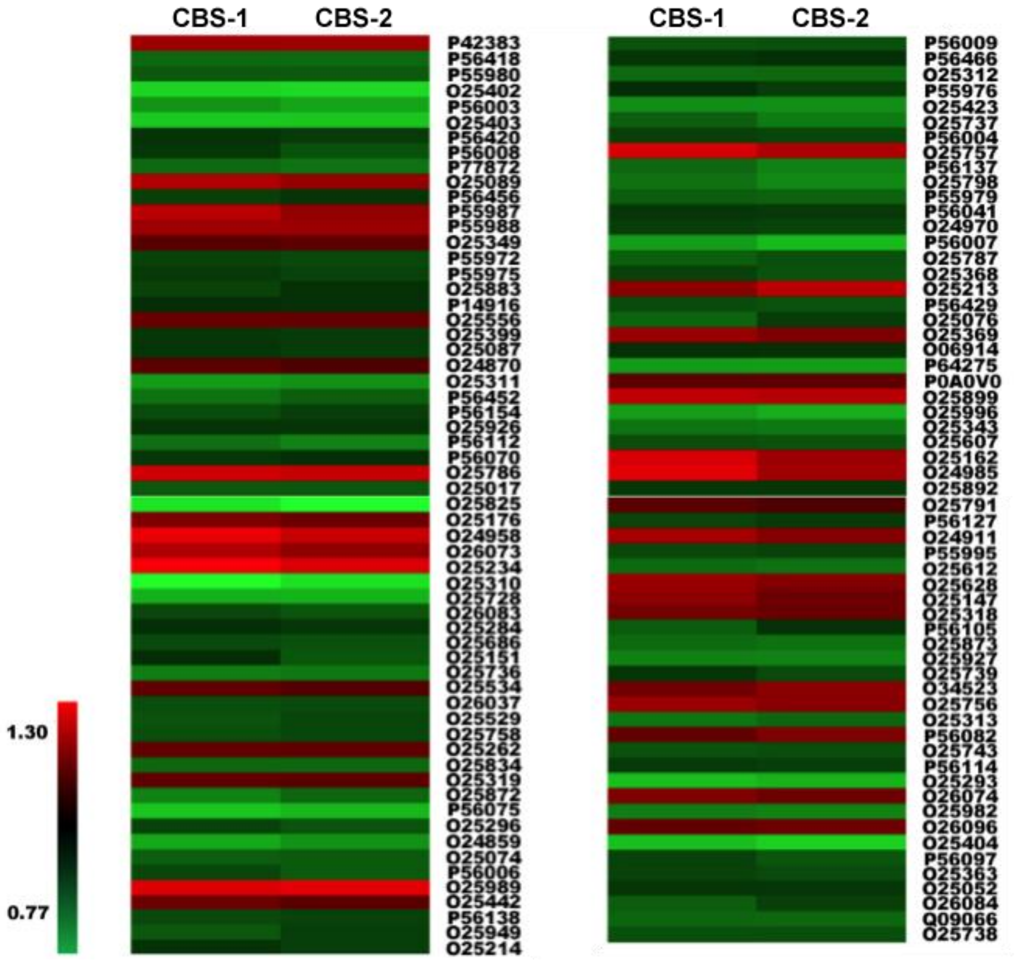

Fig. S4 Heat map of the Bi-regulated proteins in *H. pylori* 26695.Proteins are indicated by Uniprot IDs. Totally 119 proteins were identified, with a color gradient indicating the observed up- and down-regulated fold change ratios ranging from 1.30 to 0.77 (red to green). The results were obtained from biological duplicates (CBS-1 and CBS-2) compared with the untreated control group.

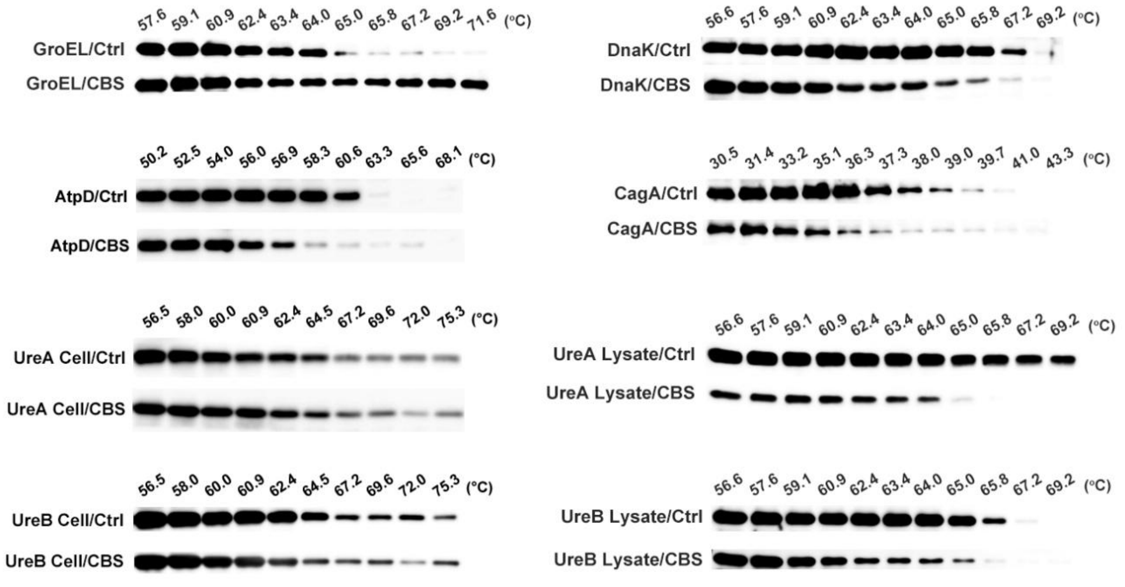

Fig. S5 Representative western blot results of CBS-targeting proteins evaluated by CETSA. To generate the protein thermal melt curves, the band intensities of soluble proteins at their corresponding lowest temperatures are set at 100%.

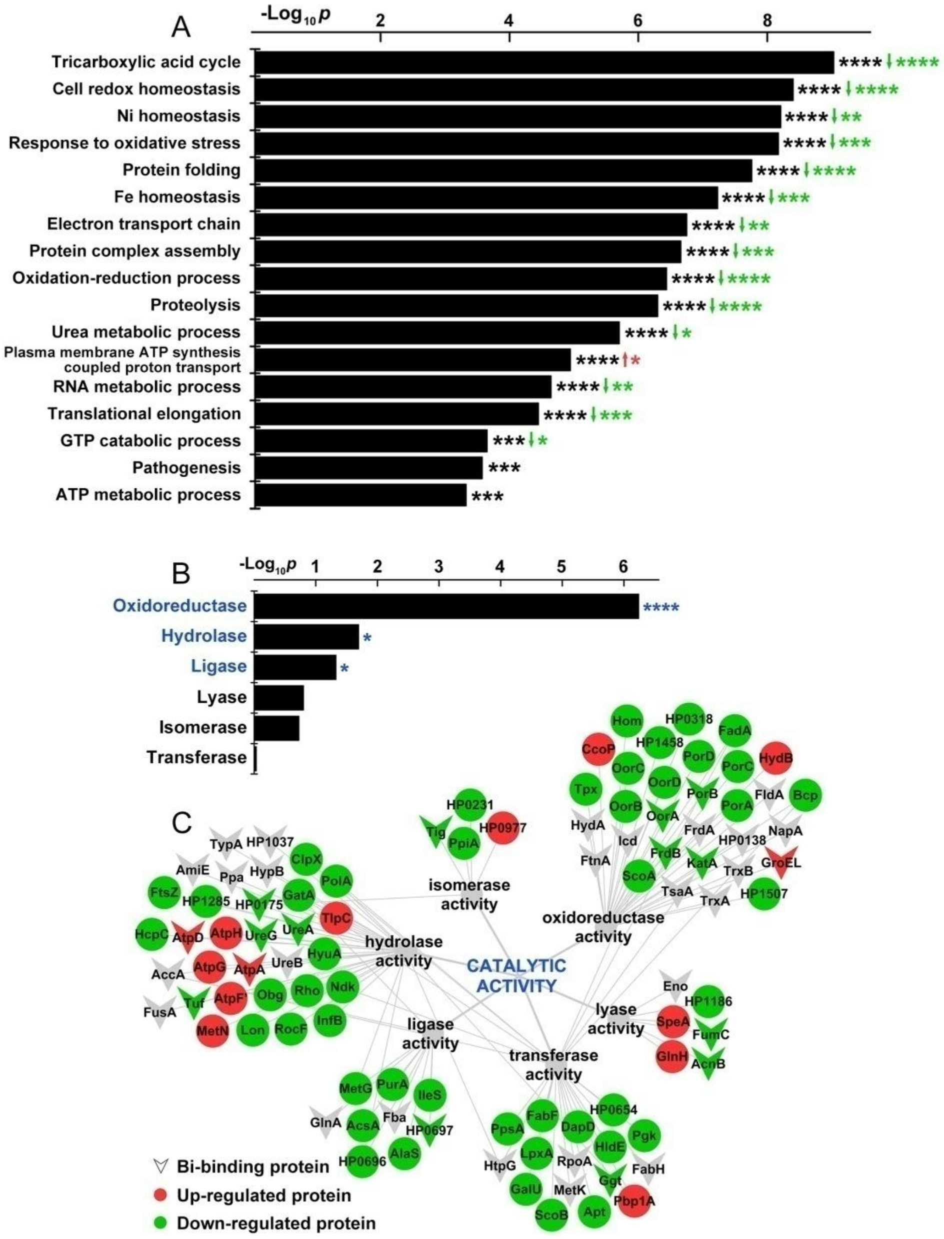

Fig. S6 Multiple pathways disrupted by bismuth drug CBS in *H. pylori*.(A) Enriched Bi-influenced functional PPI subnetworks in *H. pylori*. The enrichment *p*-values were calculated on the basis of hypergeometric distribution. Arrows indicate the up- or down-regulation of the functional categories upon Bi treatment. (B) Enrichment *p*-values of Bi-influenced catalytic activities, which indicated that *H. pylori* proteins with oxidoreductase, hydrolase and ligase activities were significantly influenced by bismuth drug. (C) Concept gene-network of Bi-associated proteins annotated with catalytic activities. The identified Bi-associated proteins are represented in different colors and shapes. (*, 0.01 <p< 0.05; **, 0.001 <p< 0.01; ***, 0.0001 <p< 0.001; ****, p< 0.0001)

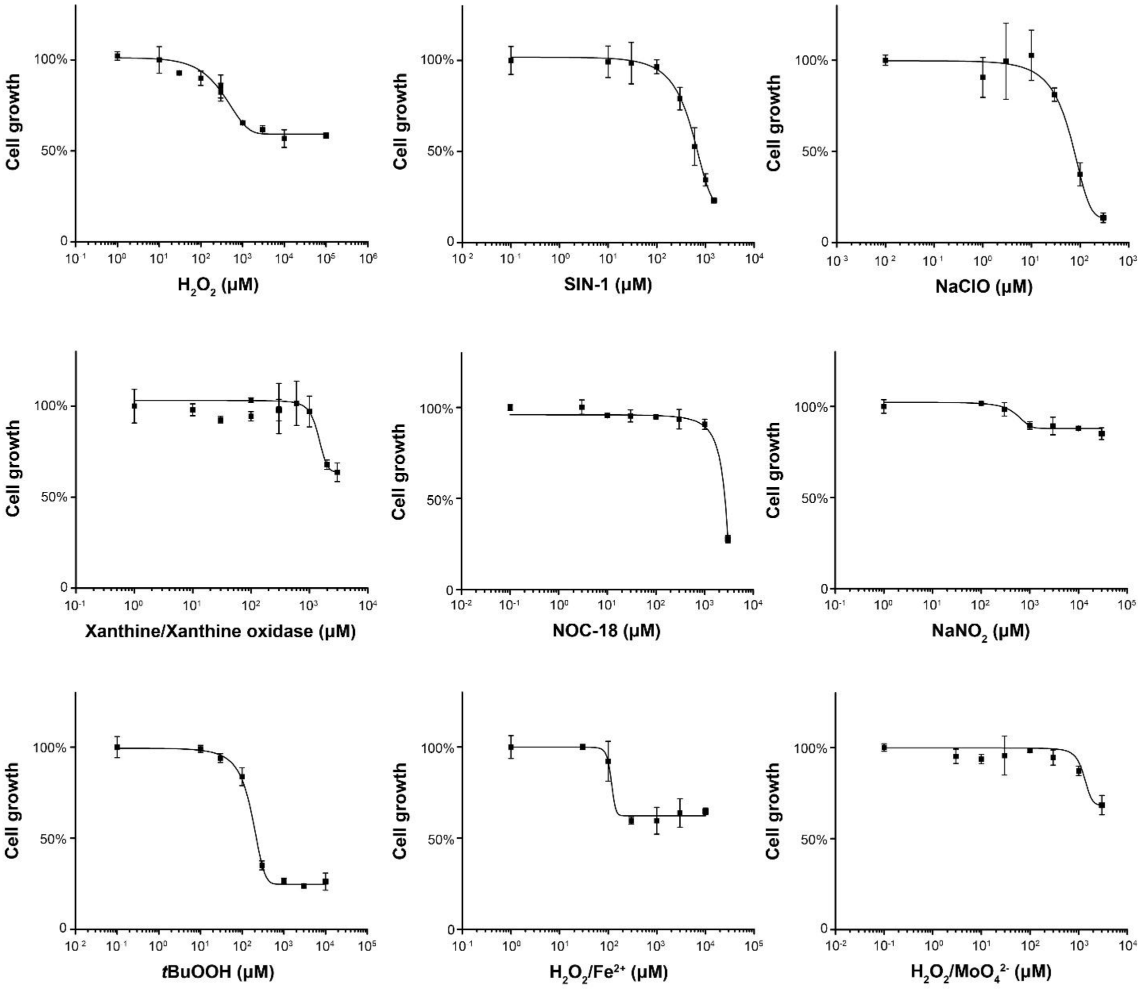

Fig. S7 Evaluation of the dose-response relationships of major ROS/RNS against the growth of *H. pylori*. The tested ROS/RNS included hydrogen peroxide (H_2O_2), peroxinitrite (SIN-1), hypochlorous acid (NaClO), superoxide (Xanthine/Xanthine oxidase), nitric oxide (NOC-18), nitrogen dioxide ($NaNO_2$), alkyl hydroperoxide (*t*BuOOH), hydroxyl radical (H_2O_2/Fe^{2+}), and singlet oxygen (H_2O_2/MoO_4^{2-}). Our data showed that *H. pylori* possessed strong defensive ability towards major ROS/RNS in certain concentrations.

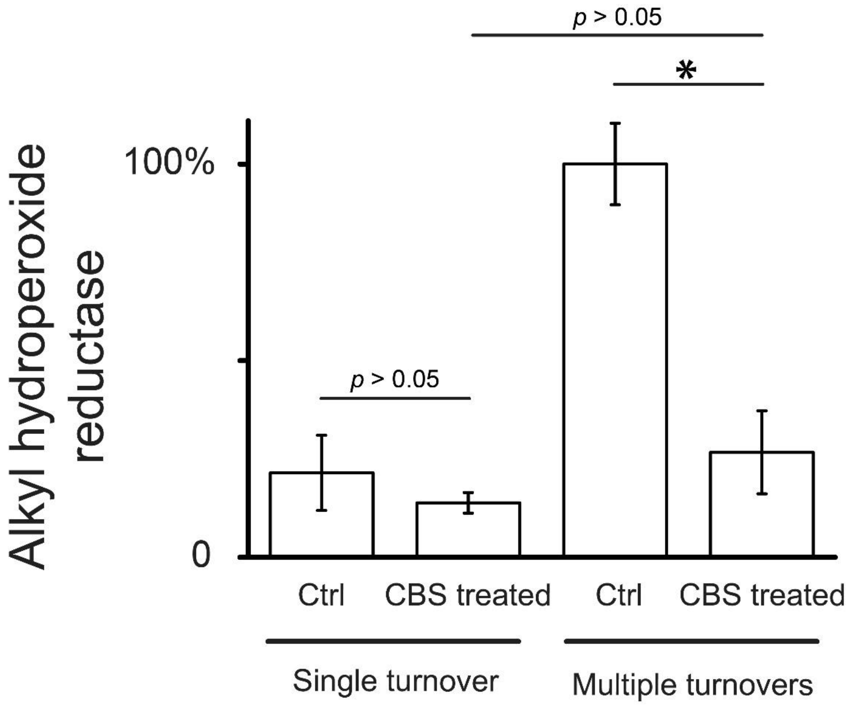

Fig. S8 The alkyl hydroperoxide reductase (AhpC) activity in *H. pylori*. When chemical reductant DTT was used for enzyme regeneration, AhpC in the control cells exhibited significantly higher activity against the alkyl radicals than in CBS-treated cells, indicating that the AhpC activity under the multiple turnover condition was significantly influenced by CBS; while without the reductant (single turnover condition), AhpC activity in control and CBS-treated cells showed insignificant differences. (* $p < 0.05$)

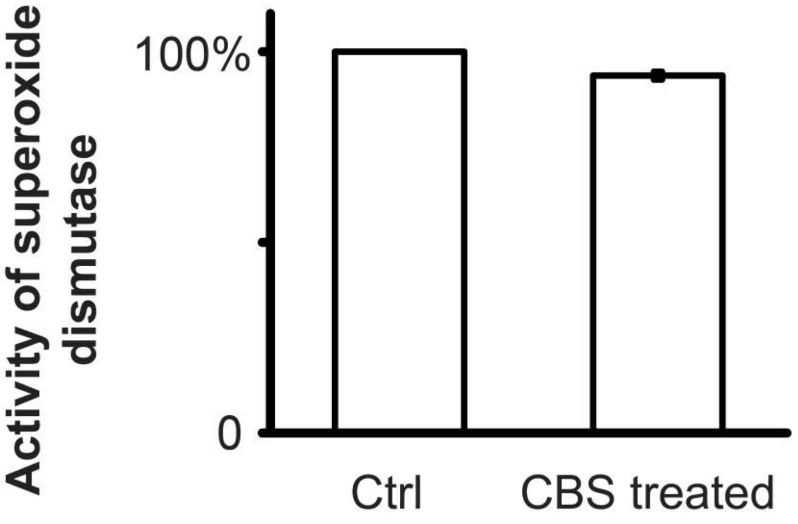

Fig. S9 The superoxide dismutase (SOD) activity in *H. pylori*. Upon CBS treatment, the activity of SOD in *H. pylori* was not significantly changed.

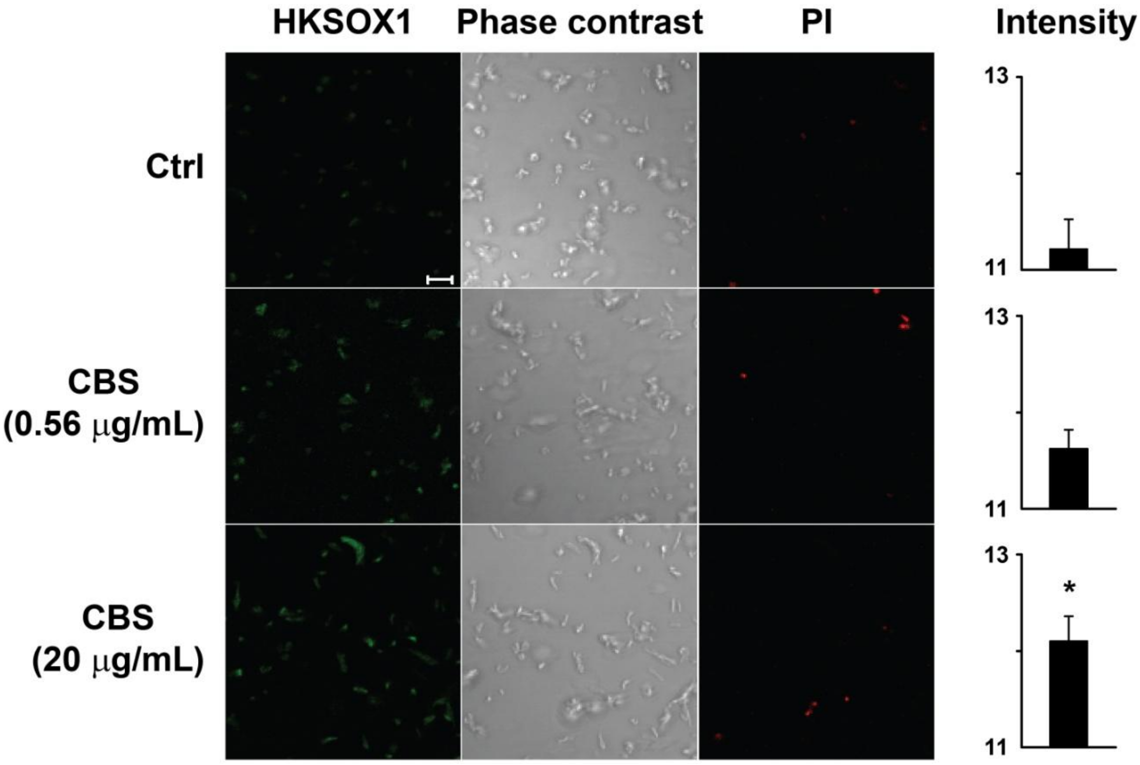

Fig. S10 Intracellular oxidative stress monitored by fluorescent probe HKSOX1.Upon treatment with CBS for 30 min, the green fluorescent intensity increased along with the concentration of CBS. The average intensity with the treatment of 20 μg/mL CBS was significantly higher than the control cells. Cell viability was indicated by propidium iodide, in which neglected red fluorescence was observed, demonstrating that the cells were alive under the experimental conditions. Such observations indicated that CBS treatment increased the accumulation of oxidative stress species in *H. pylori* cells, in accordance with the reduced oxidoreductase activities in *H. pylori* upon CBS treatment. Scale bar: 5 μm. (* $p< 0.05$)

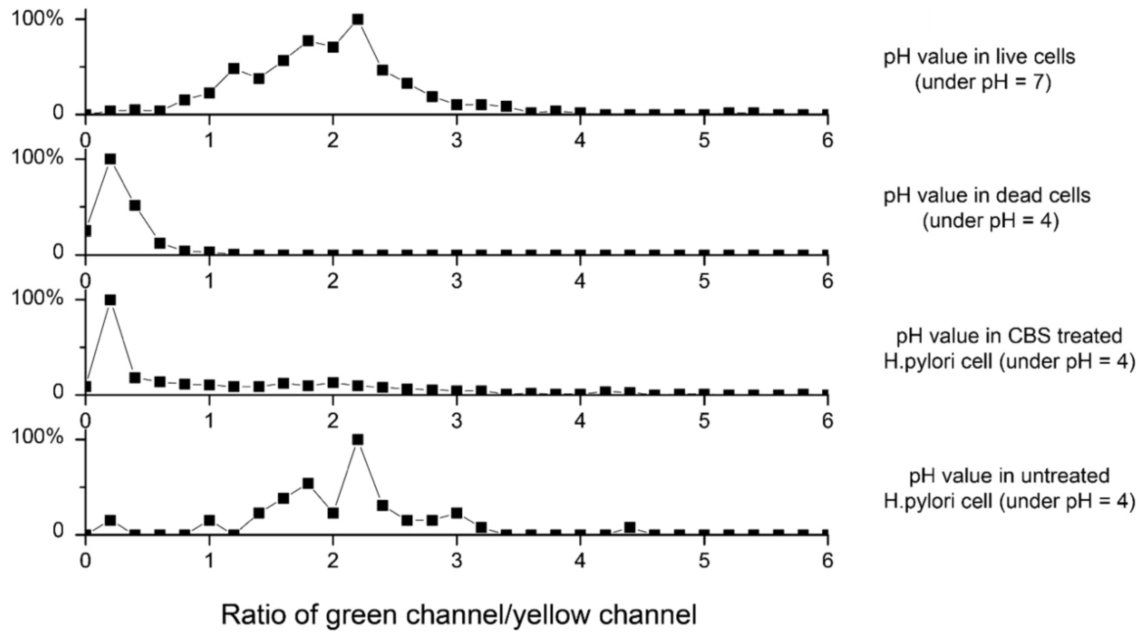

Fig. S11 Intracellular pH of live *H. pylori* cells under various conditions indicated by the fluorescent probe LysoSensor^{TM}. Ratios of the fluorescent signals from green channel over yellow channel served as the indicator. The live cells under pH 7.0 showed a broad ratio distribution ranging from 1 to 3 and centered at around 2; while the dead cells mainly focused in a relative narrow range from 0 to 0.6. Under the acidic condition (pH 4.0), *H. pylori* showed a similar ratio pattern as those under neutral condition, implying that *H. pylori* possessed the pH buffering function and had the intracellular pH kept in an optimal range. Upon CBS treatment, the pH value decreased and showed a ratio pattern similar to the dead cells, indicating that the pH buffering function was impaired by bismuth drug.

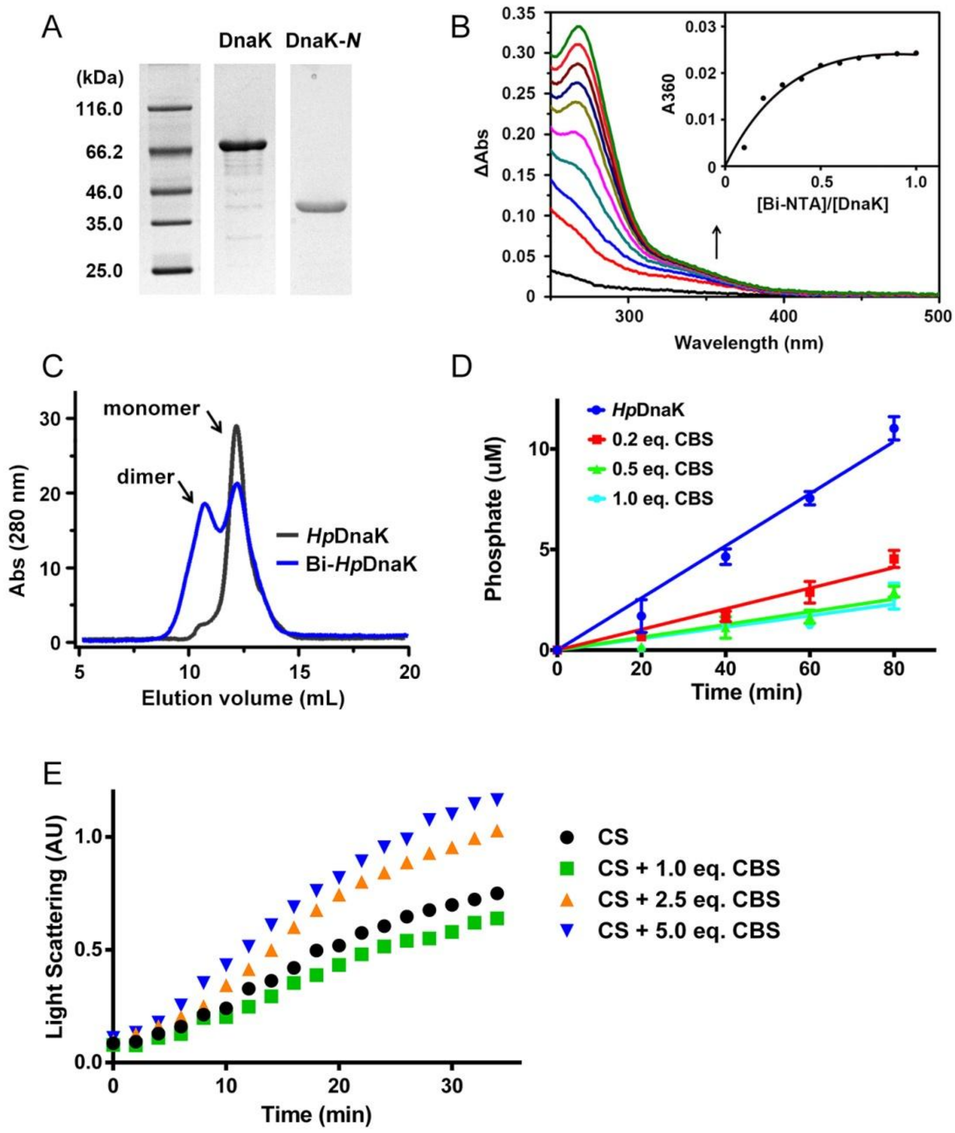

Fig. S12 (A) SDS-PAGE analysis of the purified DnaK and DnaK-*N*. (B) UV-vis difference spectra of *Hp*DnaK upon addition of different molar equivalents of Bi-NTA. The inset shows the titration curve plotted at 360 nm against different molar ratios of [Bi-NTA]/[DnaK]. The absorption at 360 nm increased with the addition of Bi-NTA and leveled off at a molar ratio of [Bi-NTA]/[DnaK] of 0.5:1, indicative of binding of 0.5 Bi^{3+} per *Hp*DnaK monomer. (C) Size-exclusion chromatography (Superdex 200 Increase 10/300 GL) profiles of *Hp*DnaK in the absence and presence of 5.0-fold CBS. (D) Time dependence of ATPase activities of apo- and Bi-bound *Hp*DnaK determined by Malachite Green method. The ATP hydrolysis rates for all samples are linear within 80 mins. (E) Influence of different concentrations of CBS on the aggregation of citrate synthase (CS) by monitoring its light scattering at 360 nm. Data show one representative result of three biological replications.

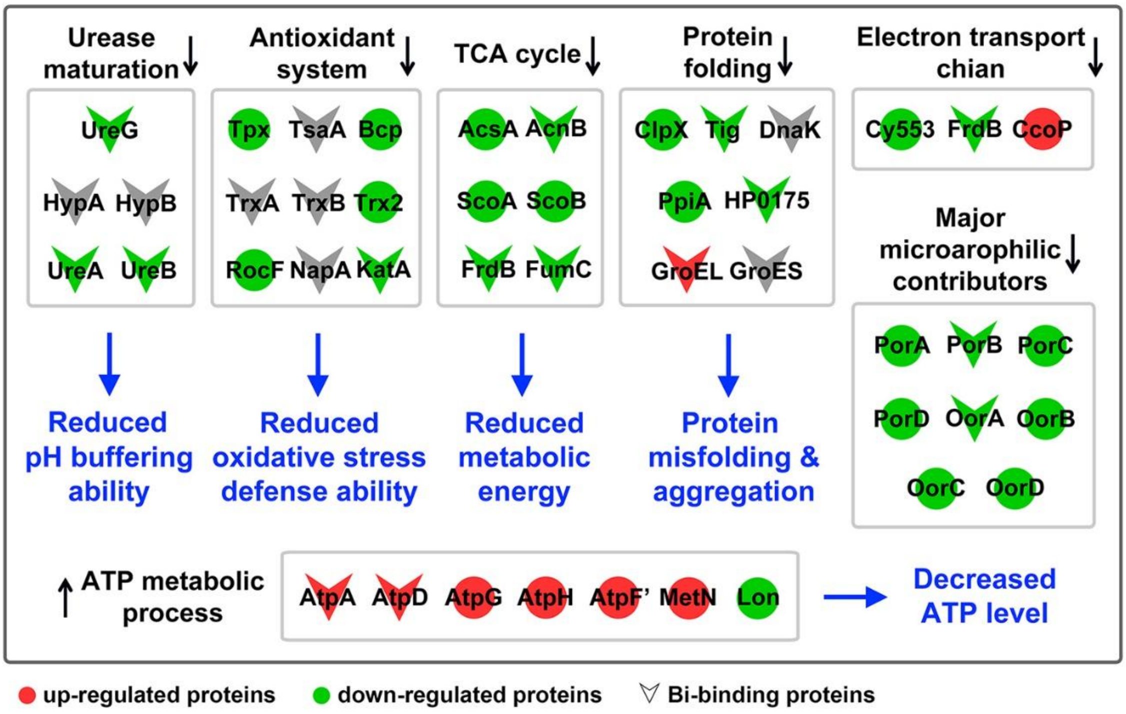

Fig. S13 Proposed biological pathways in relation to CBS-induced toxicities in *H. pylori*. According to results from the integrative metalloproteomic analyses, multiple essential functional systems/pathways in *H. pylori* were influenced by CBS. Functional alterations of the identified pathways are discussed in the supplementary text.

Table S1 Bi-binding proteins in *H. pylori*.

Locus tag	Gene name	Spot No.	Protein name	Identification approach	Ref	Associated peptides [†]
HP0010	*groEL*	6	60 kDa chaperonin	IMAC-2DE; Bi^{3+}-*TRACER*; GE-ICP-MS	15	^{58}EIELSCPVANMGAQLVK^{74}
HP0011	*groES*	46	10 kDa chaperonin	IMAC-2DE; Bi^{3+}-*TRACER*	15	^{11}LVERLEEENK^{20}<br>^{107}EHEACCHDHK^{116}
HP0025	*HP0025*[*]	–	Outer membrane protein Omp2	GE-ICP-MS	this study	
HP0027	*icd*	25	Isocitrate dehydrogenase [NADP]	Bi^{3+}-*TRACER*	this study	
HP0068	*ureG*	36	Urease accessory protein UreG	Bi^{3+}-*TRACER*	this study	^{4}IGVCGPVGSGK^{14}<br>^{58}IIGVETGGCPHTAIR^{72}
HP0072	*ureB*	5	Urease subunit beta	IMAC-2DE;<br>GE-ICP-MS; Bi^{3+}-*TRACER*	1, 15	^{23}LGDTDLIAEVEHDYTIYGEELK^{44}<br>^{241}YDVQVAIHTDTLNEAGCVEDTMA-<br>-AIAGR^{268}<br>^{269}TMHTFHTEGAGGGHAPDIIK^{288}<br>^{307}TVNTEAEHMDMLMVCHHLDK^{326}<br>^{551}EVTSKPANKVSLAQLFSIF^{569}
HP0073	*ureA*	41	Urease subunit alpha	GE-ICP-MS; Bi^{3+}-*TRACER*	1	^{133}NVGDRPVQIGSHFHFFEVNR^{152}
HP0109	*dnaK*	1	Chaperone protein DnaK	Bi^{3+}-*TRACER*	this study	^{93}IVDRNGACAIEISGK^{107}<br>^{549}TNLNENDANEIQNAINALKDCVKN-<br>-DNATK^{577}

HP0138	*HP0138*	23	Conserved hypothetical iron-sulfur protein	Bi^{3+}-*TRACER*	this study	[131]GIQAQETDLGELIIQLINEHPVHIVV-PAIHK[161]<br>[174]LNAAYEEEPEKLNAIAR[190]
HP0154	*eno*	15	Enolase	Bi^{3+}-*TRACER*	this study	[67]ACENVNSVIK[76]
HP0170	*HP0170*	14	Uncharacterized protein	Bi^{3+}-*TRACER*	this study	
HP0175	*HP0175*	–	Putative peptidyl-prolyl cis-trans isomerase HP0175	GE-ICP-MS	1	[63]QRNPNFDFDKLKEKEKEALIDQAIR[87]
HP0176	*fba*	29	Fructose-bisphosphate aldolase	Bi^{3+}-*TRACER*	this study	[88]ESCEKAVKAGF[98]
HP0177	*efp*	35	Elongation factor P	Bi^{3+}-*TRACER*	this study	[54]TFHAGDKCEEPNLVEK[69]
HP0191	*frdB*	33	Fumarate reductase iron-sulfur subunit	Bi^{3+}-*TRACER*	this study	
HP0192	*frdA*	8	Fumarate reductase flavoprotein subunit	Bi^{3+}-*TRACER*	this study	
HP0197	*metK*	19	S-adenosylmethionine synthase	Bi^{3+}-*TRACER*	this study	[32]IIERDQKAKVACETL[46]<br>[127]ACKETETL[134]<br>[253]GGSCPHGGGAF[263]<br>[264]SGKDPSKVDRSAAY[277]<br>[285]NLVASGVCDK[294]<br>[322]SSAELEKCVKSVF[334]<br>[369]TWEKTNKAEEIKAFF[383]
HP0202	*fabH*	32	3-oxoacyl-[acyl-carrier-protein] synthase 3	Bi^{3+}-*TRACER*	this study	

HP0210	*htpG*	4	Chaperone protein HtpG	Bi^{3+}-*TRACER*	this study	^{157}GKFEISECVK^{166}<br>^{562}TLELNPNHAILQK^{574}
HP0243	*napA*	43	DNA protection during starvation protein	IMAC-2DE; Bi^{3+}-*TRACER*; GE-ICP-MS	15	
HP0254	*HP0254**	–	Outer membrane protein Omp8	GE-ICP-MS	this study	
HP0294	*amiE*	22	Aliphatic amidase	Bi^{3+}-*TRACER*	this study	
HP0480	*typA*	3	GTP-binding protein TypA / BipA homolog	Bi^{3+}-*TRACER*	this study	
HP0486	*HP0486**	–	Uncharacterized protein	GE-ICP-MS	this study	
HP0512	*glnA*	16	Glutamine synthetase	Bi^{3+}-*TRACER*	this study	
HP0557	*accA*	30	Acetyl-coenzyme A carboxylase carboxyl transferase subunit alpha	Bi^{3+}-*TRACER*	this study	
HP0589	*HP0589*	20	Ferredoxin oxidoreductase, alpha subunit	Bi^{3+}-*TRACER*	this study	^{15}AAIEVGCR^{22}
HP0599	*HP0599*	17	Hemolysin secretion protein	Bi^{3+}-*TRACER*	this study	^{24}EVNLYQSLLNLCLHEGFVGIK^{44}<br>^{100}NTSCVGEYHK^{109}<br>^{306}VFCGLAKLDHVVFK^{319}<br>^{320}NNLYGMVFGLNSFDITSHKNCR^{341}
HP0604	*hemE*	–	Uroporphyrinogen decarboxylase	GE-ICP-MS	this study	
HP0620	*ppa*	37	Inorganic pyrophosphatase	Bi^{3+}-*TRACER*	this study	

HP0630	*HP0630*	–	Modulator of drug activity Mda66	GE-ICP-MS	this study	
HP0631	*HP0631*	26	Quinone-reactive Ni/Fe hydrogenase, small subunit	Bi^{3+}-*TRACER*	this study	^{262}RGHFDAGEFVEHFGDENAK^{280}
HP0653	*ftnA*	42	Bacterial non-heme ferritin	Bi^{3+}-*TRACER*	this study	
HP0697	*HP0697*	38	Uncharacterized protein	Bi^{3+}-*TRACER*	this study	
HP0706	*HP0706*[*]	–	Outer membrane protein Omp15	GE-ICP-MS	this study	
HP0779	*acnB*	7	Aconitase hydratase B	Bi^{3+}-*TRACER*	this study	^{696}RPHAIDEVFIGSCMTNIGHFR^{716}
HP0795	*tig*	10	Trigger factor	Bi^{3+}-*TRACER*	this study	^{124}ECVPSVGVEVPNEEK^{138}
HP0824	*trxA*	44	Thioredoxin	Bi^{3+}-*TRACER*; GE-ICP-MS	this study	^{53}ICKVNTDEQEELSAK^{67}
HP0825	*trxB*	28	Thioredoxin reductase	Bi^{3+}-*TRACER*	this study	
HP0875	*katA*	24	Catalase	Bi^{3+}-*TRACER*; GE-ICP-MS	this study	
HP0900	*hypB*	34	Hydrogenase/urease nickel incorporation protein HypB	Bi^{3+}-*TRACER*	this study	^{74}FCVVEGDLQTNR^{85}<br>^{174}MCADVIISK^{183}
HP0958	*HP0958*	31	Uncharacterized protein	Bi^{3+}-*TRACER*	this study	^{162}KKKEELVEKTEPKIY^{176}
HP1037	*HP1037*	18	Aminopeptidase	Bi^{3+}-*TRACER*	this study	
HP1111	*HP1111*	27	Pyruvate ferredoxin oxidoreductase, beta subunit	Bi^{3+}-*TRACER*	this study	

HP1118	*HP1118*	40	Gamma-glutamyltranspeptidaseGgt	Bi^{3+}-*TRACER*	this study	
HP1132	*atpD*	11	ATP synthase subunit beta	Bi^{3+}-*TRACER*	this study	
HP1134	*atpA*	9	ATP synthase subunit alpha	Bi^{3+}-*TRACER*	this study	
HP1161	*fldA*	44	Flavodoxin	Bi^{3+}-*TRACER*	this study	
HP1177	*HP1177*[*]	–	Outer membrane protein Omp27	GE-ICP-MS	this study	
HP1195	*fusA*	2	Elongation factor G	Bi^{3+}-*TRACER*	this study	^{40}IGEVHDGAATMDWMEQEKER^{59} ^{382}DTLTGDTLCDEK^{393}
HP1199	*rplL*	–	50S ribosomal protein L7/L12	GE-ICP-MS	this study	
HP1205	*tuf*	12	Elongation factor Tu	IMAC-2DE; GE-ICP-MS; Bi^{3+}-*TRACER*	1, 15	^{46}DYDNIDNAPEEKER^{59} ^{60}GITIATSHIEYETENR^{75} ^{76}HYAHVDCPGHADYVK^{90} ^{178}ALEEAKAGNVGEWGEK^{193} ^{295}GMVLCKPGSITPHK^{308}
HP1286	*HP1286*	–	Conserved hypothetical secreted protein	GE-ICP-MS	1	
HP1293	*rpoA*	13	DNA-directed RNA polymerase subunit alpha	Bi^{3+}-*TRACER*; GE-ICP-MS	this study	^{270}CFNCLDKIGIK^{280}
HP1325	*fumC*	–	Fumarate hydratase class II	IMAC-2DE	15	^{297}LASGPRCGLGEL^{308} ^{383}NIHCASGIEPNREKIDY^{399}

HP1376	*fabZ*	–	3-hydroxyacyl-[acyl-carrier-protein] dehydratase FabZ	GE-ICP-MS	this study	
HP1456	*lpp20*[*]	–	LPP20 lipoprotein	GE-ICP-MS	this study	
HP1469	*HP1469*[*]	–	Outer membrane protein Omp31	GE-ICP-MS	this study	
HP1501	*HP1501*[*]	–	Outer membrane protein Omp32	GE-ICP-MS	this study	
HP1512	*HP1512*[*]	–	Iron-regulated outer membrane protein FrpB	GE-ICP-MS	this study	
HP1555	*tsf*	21	Elongation factor Ts	Bi^{3+}-*TRACER*	this study	^{14}DLTDAGMMDCK^{24}<br>^{309}TIAQVVADCSKEWNDDLK^{326}<br>^{213}ETLALIAEIEKDNEEAKR^{230}
HP1562	*HP1562*	–	Iron(III) ABC transporter, periplasmic iron-binding protein CeuE	GE-ICP-MS	1	
HP1563	*tsaA*	39	Probable peroxiredoxin	IMAC-2DE; GE-ICP-MS; Bi^{3+}-*TRACER*	1, 15	^{137}HAVINDLPLGR^{147}<br>^{161}HFEEHGEVCPAGW^{173}<br>^{182}ATHQGVAEYLK^{192}

† Bi-associated peptides identified by Bi-IMAC.[16]

* Membrane proteins identified by GE-ICP-MS in this study.

Table S2 PMF (peptide mass fingerprinting) results of newly identified Bi-binding proteins in *H. pylori*.

Protein name	Accession No.	Locus tag	Protein score	Protein score C.I.%	Protein MW (Da)	Theoretical pI	Peptidecounts
Outer membrane protein (Omp2)	gi|15644658	HP0025	206	100	77959.0	9.2	18
Isocitrate dehydrogenase [NADP] (Icd)	gi|446246133	HP0027	511	100	47728.6	7.6	25
Urease accessory protein UreG	gi|446160907	HP0068	266	100	22112.2	5.0	11
Molecular chaperone DnaK	gi|15644739	HP0109	560	100	67124.8	5.0	27
Conserved hypothetical iron-sulfur protein	gi|446339449	HP0138	410	100	54829.1	6.6	17
Enolase (Eno)	gi|446878417	HP0154	492	100	46619.1	5.5	20
Uncharacterized protein (HP0170)	gi|446113171	HP0170	437	100	28661.1	4.6	11
Fructose-bisphosphate aldolase (Fba)	gi|446883215	HP0176	391	100	33865.4	5.9	20
Elongation factor P (Efp)	gi|446897009	HP0177	266	100	20831.7	5.4	12
Fumarate reductase iron-sulfur subunit (FrdB)	gi|447205183	HP0191	436	100	28317.7	5.3	14
Fumarate reductase flavoprotein subunit (FrdA)	gi|446628675	HP0192	461	100	80754.7	6.9	31
S-adenosylmethionine synthase (MetK)	gi|446577832	HP0197	513	100	42677.7	6.0	23
3-oxoacyl-[acyl-carrier-protein] synthase 3 (FabH)	gi|446319931	HP0202	193	100	36730.9	5.7	10
Chaperone protein HtpG	gi|445992752	HP0210	658	100	71400.6	5.4	27
Outer membrane protein (Omp8)	gi|15644882	HP0254	225	100	47526.1	9.2	13
Aliphatic amidase (AmiE)	gi|447138473	HP0294	332	100	38315.9	6.2	15
GTP-binding protein TypA / BipA homolog (TypA)	gi|446712877	HP0480	630	100	66805.3	5.3	30
Uncharacterized protein (HP0486)	gi|15645113	HP0486	381	100	59492.7	9.4	18

Glutamine synthetase (GlnA)	gi|446559804	HP0512	588	100	54764.1	5.8	24
Acetyl-coenzyme A carboxylase carboxyl transferase subunit alpha (AccA)	gi|446234172	HP0557	244	100	35030.1	5.4	10
Ferredoxin oxidoreductase, alpha subunit (OorA)	gi|447129600	HP0589	348	100	41596.4	6.0	18
Hemolysin secretion protein (HylB)	gi|446389947	HP0599	569	100	48616.4	5.8	25
Uroporphyrinogen decarbocylase (HemE)	gi|15645229	HP0604	124	100	38617.3	8.2	8
Inorganic pyrophosphatase (Ppa)	gi|446970117	HP0620	88	100	19316.9	5.0	5
Modulator of drug activity (Mda66)	gi|15645254	HP0630	200	100	21648.0	6.6	5
Quinone-reactive Ni/Fe hydrogenase, small subunit (HydA)	gi|446421172	HP0631	228	100	42954.2	6.4	11
Bacterial non-heme ferritin (FtnA)	gi|446871946	HP0653	320	100	19330.6	5.4	6
Uncharacterized protein	gi|487788862	HP0697	110	100	19956.9	5.2	6
Outer membrane protein (Omp15)	gi|15645329	HP0706	423	100	30152.1	9.1	8
Aconitase hydratase B (AcnB)	gi|487789973	HP0779	526	100	93345.1	6.2	35
Trigger factor (Tig)	gi|446970475	HP0795	419	100	52013.2	5.3	29
Thioredoxin(TrxA)	gi|445942344	HP0824	536	100	12018.2	5.2	13
Thioredoxin reductase (TrxB)	gi|446486566	HP0825	373	100	34030.0	5.9	14
Catalase (KatA)	sp|P77872	HP0875	153	100	58706.1	8.7	15
Hydrogenase/urease nickel incorporation protein HypB	gi|445925767	HP0900	284	100	27310.4	5.4	13
Uncharacterized protein	gi|447014579	HP0958	153	100	29901.6	5.7	13
Aminopeptidase	gi|446599828	HP1037	407	100	40941.9	5.8	18
Pyruvate ferredoxin oxidoreductase, beta	gi|446160280	HP1111	221	100	35389.7	8.3	7

subunit (PorB)							
Gamma-glutamyltranspeptidase (Ggt)	gi|447177215	HP1118	158	100	61112.9	9.3	8
ATP synthase subunit beta (AtpD)	gi|446569020	HP1132	391	100	51502.6	5.3	25
ATP synthase subunit alpha (AtpA)	gi|446002651	HP1134	532	100	55279.9	5.3	29
Flavodoxin (FldA)	gi|446438223	HP1161	436	100	17481.7	4.4	8
Outer membrane protein (Omp27)	gi|15645791	HP1177	826	100	69991.0	9.2	25
Elongation factor G (FusA)	gi|446023959	HP1195	544	100	77314.3	5.2	32
DNA-directed RNA polymerase subunit alpha (RpoA)	gi|446787291	HP1293	363	100	38588.9	5.0	20
3-hydroxyacyl-[acyl-carrier-protein] dehydratase FabZ	gi|108563747	HP1376	178	100	18156.5	6.6	9
Membrane associated protein Lpp20	gi|14210190	HP1456	324	100	19197.2	9.5	8
Outer membrane protein (Omp31)	gi|15646078	HP1469	322	100	28131.3	9.5	11
Outer membrane protein (Omp32)	gi|15646110	HP1501	214	100	43125.2	9.5	10
Iron-regulated outer membrane protein (FrpB)	gi|15646121	HP1512	321	100	97495.3	9.0	23
Elongation factor Ts (Tsf)	gi|445936542	HP1555	633	100	39841.1	6.2	27

Table S3 Differentially expressed (34 up- and 85 down-regulated) proteins in Bi-treated *H. pylori*.

**(*Gene name*) Protein name**	**Locus tag**	**Fold change (treated/ctrl)**	**Directly annotated GO terms**
Oxidation-reduction process			
(*HP0632*) Quinone-reactive Ni/Fe hydrogenase, large subunitHydB	HP0632	1.37 ± 0.02	**Process**:GO:0055114 (oxidation-reduction process). **Function**:GO:0008901 (ferredoxin hydrogenase activity), GO:0016151 (nickel cation binding), GO:0047067 (hydrogen:quinone oxidoreductase activity).
(*HP1227*) Cytochrome c-553 Cy553	HP1227	0.33 ± 0.03	**Process**:GO:0022900 (electron transport chain), GO:0055114 (oxidation-reduction process). **Function**:GO:0005506 (iron ion binding), GO:0009055 (electron carrier activity), GO:0020037 (heme binding). **Component**: GO:0042597 (periplasmic space).
(*HP0589*) 2-oxoglutarate-acceptor oxidoreductase subunitOorA	HP0589	0.49 ± 0.02	**Process**:GO:0055114 (oxidation-reduction process). **Function**:GO:0016491 (oxidoreductase activity), GO:0047553 (2-oxoglutarate synthase activity).
(*HP0590*) Ferredoxin oxidoreductase, beta subunitOorB	HP0590	0.57 ± 0.01	**Process**:GO:0008152 (metabolic process), GO:0055114 (oxidation-reduction process). **Function**:GO:0003824 (catalytic activity), GO:0030976 (thiamine pyrophosphate binding), GO:0047553 (2-oxoglutarate synthase activity).
(*HP0591*) Ferredoxin oxidoreductase, gamma subunitOorC	HP0591	0.55 ± 0.03	**Process**:GO:0055114 (oxidation-reduction process). **Function**:GO:0016903 (oxidoreductase activity, acting on the aldehyde or oxo group of donors).
(*HP0588*) Ferrodoxin-like proteinOorD	HP0588	0.33 ± 0.04	**Process**:GO:0055114 (oxidation-reduction process). **Function**:GO:0009055 (electron carrier activity), GO:0046872 (metal ion binding), GO:0047553 (2-oxoglutarate synthase activity), GO:0051536 (iron-sulfur cluster binding), GO:0051539 (4 iron, 4 sulfur cluster binding).

(*HP1110*) Pyruvate ferredoxin oxidoreductase, alpha subunitPorA	HP1110	0.64 ± 0.02	**Process**:GO:0055114 (oxidation-reduction process). **Function**:GO:0016491 (oxidoreductase activity), GO:0019164 (pyruvate synthase activity).
(*HP1111*) Pyruvate ferredoxin oxidoreductase, beta subunitPorB	HP1111	0.70 ± 0.05	**Process**:GO:0008152 (metabolic process), GO:0055114 (oxidation-reduction process). **Function**:GO:0003824 (catalytic activity), GO:0019164 (pyruvate synthase activity), GO:0030976 (thiamine pyrophosphate binding).
(*HP1108*) Pyruvate ferredoxin oxidoreductase, gamma subunitPorC	HP1108	0.53 ± 0.01	**Process**:GO:0055114 (oxidation-reduction process). **Function**:GO:0016625 (oxidoreductase activity, acting on the aldehyde or oxo group of donors, iron-sulfur protein as acceptor), GO:0019164 (pyruvate synthase activity).
(*HP1109*) Pyruvate ferredoxin oxidoreductase, delta subunit PorD	HP1109	0.58 ± 0.04	**Process**:GO:0055114 (oxidation-reduction process). **Function**:GO:0009055 (electron carrier activity), GO:0016625 (oxidoreductase activity, acting on the aldehyde or oxo group of donors, iron-sulfur protein as acceptor), GO:0019164 (pyruvate synthase activity), GO:0046872 (metal ion binding), GO:0051539 (4 iron, 4 sulfur cluster binding).
(*ccoP*) Cbb3-type cytochrome c oxidase subunit †	HP0147	1.95 ± 0.12	**Process:**GO:0006119(oxidative phosphorylation),GO:0015992(proton transport), GO:0022900 (electron transport chain). **Function**:GO:0009055(electron carrier activity), GO:0016491 (oxidoreductase activity), GO:0020037 (heme binding), GO:0046872 (metal ion binding). **Component**: GO:0005886 (plasma membrane), GO:0016020 (membrane), GO:0016021 (integral component of membrane), GO:0070469 (respiratory chain).
(*HP1458*) Thioredoxin	HP1458	0.40 ± 0.05	**Process**:GO:0006662 (glycerol ether metabolic process), GO:0045454 (cell redox homeostasis). **Function**:GO:0009055 (electron carrier activity), GO:0015035 (protein disulfide oxidoreductase activity).

(*scoA*) Succinyl-CoA:3-ketoacid coenzyme A transferase subunit A	HP0691	0.64 ± 0.05	**Process**:GO:0006091 (generation of precursor metabolites and energy), GO:0006099 (tricarboxylic acid cycle), GO:0008152 (metabolic process). **Function**:GO:0004318 (enoyl-[acyl-carrier-protein] reductase (NADH) activity), GO:0004450 (isocitrate dehydrogenase (NADP+) activity), GO:0008260 (3-oxoacid CoA-transferase activity).
(*scoB*) Succinyl-CoA:3-ketoacid coenzyme A transferase subunit B	HP0692	0.44 ± 0.04	**Process**:GO:0006099 (tricarboxylic acid cycle), GO:0008152 (metabolic process). **Function**:GO:0008260 (3-oxoacid CoA-transferase activity).
(*bcp*) Putative peroxiredoxin	HP0136	0.57 ± 0.05	**Process**:GO:0045454 (cell redox homeostasis), GO:0055114 (oxidation-reduction process). **Function**:GO:0004601 (peroxidase activity), GO:0008379 (thioredoxin peroxidase activity), GO:0051920 (peroxiredoxin activity).
(*katA*) Catalase †	HP0875	0.60 ± 0.04	**Process**:GO:0019439 (aromatic compound catabolic process), GO:0042744 (hydrogen peroxide catabolic process), GO:0044248 (cellular catabolic process), GO:0046677 (response to antibiotic), GO:0055114 (oxidation-reduction process). **Function**:GO:0004096 (catalase activity), GO:0016407 (acetyltransferase activity), GO:0020037 (heme binding), GO:0046872 (metal ion binding). **Component**:GO:0005737 (cytoplasm).
(*tpx*) Probable thiol peroxidase †	HP0390	0.69 ± 0.10	**Process**:GO:0045454 (cell redox homeostasis), GO:0050896 (response to stimulus), GO:0055114 (oxidation-reduction process). **Function**:GO:0008379 (thioredoxin peroxidase activity).
(*acnB*) Aconitate hydratase 2	HP0779	0.60 ± 0.03	**Process**:GO:0006099 (tricarboxylic acid cycle). **Function**:GO:0046872 (metal ion binding), GO:0047456 (2-methylisocitrate dehydratase activity), GO:0051539 (4 iron, 4 sulfur cluster binding), GO:0052632 (citrate hydro-lyase (cis-aconitate-forming) activity), GO:0052633 (isocitrate hydro-lyase (cis-aconitate-forming) activity). **Component**:GO:0005829 (cytosol).

(*acsA*) Acetyl-coenzyme A synthetase[†]	HP1045	0.65 ± 0.01	**Process**:GO:0006090 (pyruvate metabolic process), GO:0006099 (tricarboxylic acid cycle),GO:0008152 (metabolic process), GO:0019427 (acetyl-CoA biosynthetic process from acetate), GO:0019541 (propionate metabolic process), GO:0019643 (reductive tricarboxylic acid cycle), GO:0044248 (cellular catabolic process). **Function**:GO:0003987 (acetate-CoA ligase activity), GO:0004823 (leucine-tRNA ligase activity), GO:0005524 (ATP binding), GO:0016208 (AMP binding), GO:0046872 (metal ion binding).
(*HP1507*) Conserved hypothetical ATP-binding protein	HP1507	0.65 ± 0.01	**Process**:GO:0055114 (oxidation-reduction process). **Function**:GO:0004754 (saccharopine dehydrogenase (NAD+, L-lysine-forming) activity), GO:0005524 (ATP binding), GO:0016491 (oxidoreductase activity).
(*HP0318*) Putative uncharacterized protein[†]	HP0318	0.71 ± 0.03	**Process**:GO:0042823 (pyridoxal phosphate biosynthetic process), GO:0055114 (oxidation-reduction process). **Function**:GO:0004733 (pyridoxamine-phosphate oxidase activity), GO:0010181 (FMN binding).
(*fumC*) Fumarate hydratase class II	HP1325	0.72 ± 0.05	**Process**:GO:0006099 (tricarboxylic acid cycle), GO:0006106 (fumarate metabolic process). **Function**:GO:0004333 (fumarate hydratase activity). **Component**:GO:0045239 (tricarboxylic acid cycle enzyme complex).
(*frdB*) Fumarate reductase iron-sulfur subunit	HP0191	0.75 ± 0.01	**Process**:GO:0006099 (tricarboxylic acid cycle), GO:0022900 (electron transport chain). **Function**:GO:0000104 (succinate dehydrogenase activity), GO:0009055 (electron carrier activity), GO:0046872 (metal ion binding), GO:0051537 (2 iron, 2 sulfur cluster binding), GO:0051538 (3 iron, 4 sulfur cluster binding), GO:0051539 (4 iron, 4 sulfur cluster binding).
Protein transport / metabolic process			
(*groEL*) 60 kDa chaperonin; GroEL protein [†]	HP0010	1.59 ± 0.05	**Process**:GO:0006950(response to stress),GO:0042026 (protein refolding). **Function**:GO:0005524(ATP binding),GO:0016491 (oxidoreductase activity), GO:0051082 (unfolded protein binding). **Component**:GO:0005737(cytoplasm).

(*tuf*) Elongation factor Tu; EF-Tu†	HP1205	0.46 ± 0.02	**Process**:GO:0006184 (GTP catabolic process), GO:0006414 (translational elongation). **Function**:GO:0003746 (translation elongation factor activity), GO:0003924 (GTPase activity), GO:0005525 (GTP binding). **Component**:GO:0005737 (cytoplasm).
(*ppiA*) Peptidyl-prolyl cis-trans isomerase	HP1441	0.52 ± 0.01	**Process**:GO:0000413 (protein peptidyl-prolyl isomerization), GO:0006457 (protein folding), GO:0044267 (cellular protein metabolic process). **Function**:GO:0003755 (peptidyl-prolyl cis-trans isomerase activity).
(*HP0175*) Putative peptidyl-prolyl cis-trans isomerase	HP0175	0.54 ± 0.02	**Process**:GO:0000413 (protein peptidyl-prolyl isomerization), GO:0006457 (protein folding), GO:0006508 (proteolysis). **Function**:GO:0003755 (peptidyl-prolyl cis-trans isomerase activity), GO:0008233 (peptidase activity).
(*tsf*) Elongation factor Ts; EF-Ts	HP1555	0.71 ± 0.02	**Process**:GO:0006414 (translational elongation). **Function**:GO:0003746 (translation elongation factor activity). **Component**:GO:0005737 (cytoplasm).
(*rpsB*) 30S ribosomal protein S2	HP1554	0.67 ± 0.05	**Process**:GO:0006412 (translation). **Function**:GO:0003735 (structural constituent of ribosome). **Component**:GO:0015935 (small ribosomal subunit).
(*efp*) Elongation factor P	HP0177	0.68 ± 0.02	**Process**:GO:0006412 (translation), GO:0006414 (translational elongation), GO:0043043 (peptide biosynthetic process). **Function**:GO:0003746 (translation elongation factor activity), GO:0043022 (ribosome binding). **Component**:GO:0005737 (cytoplasm).
(*rpsA*) 30S ribosomal protein S1	HP0399	0.68 ± 0.06	**Process**:GO:0006412 (translation). **Function**:GO:0003723 (RNA binding),GO:0003735 (structural constituent of ribosome). **Component**:GO:0005840 (ribosome).

(*metG*) Methionine--tRNA ligase †	HP0417	0.69 ± 0.02	**Process**:GO:0006431 (methionyl-tRNAaminoacylation), GO:0006464 (cellular protein modification process), GO:0044267 (cellular protein metabolic process). **Function**:GO:0000049 (tRNA binding),GO:0004825 (methionine-tRNA ligase activity), GO:0005524 (ATP binding), GO:0008463 (formylmethioninedeformylase activity), GO:0046872 (metal ion binding). **Component**:GO:0005737 (cytoplasm).
(*HP0977*) Conserved hypothetical secreted protein	HP0977	1.56 ± 0.06	**Process**:GO:0000413 (protein peptidyl-prolyl isomerization). **Function**:GO:0003755 (peptidyl-prolyl cis-trans isomerase activity), GO:0016853 (isomerase activity).
(*rplP*) 50S ribosomal protein L16	HP1312	0.72 ± 0.01	**Process**:GO:0006412 (translation). **Function**:GO:0000049 (tRNA binding), GO:0003735 (structural constituent of ribosome), GO:0019843 (rRNA binding). **Component**:GO:0005840 (ribosome).
(*gatA*) Glutamyl-tRNAamidotransferase subunit A †	HP0830	0.71 ± 0.01	**Process**:GO:0006412 (translation), GO:0006424 (glutamyl-tRNAaminoacylation), GO:0044267 (cellular protein metabolic process). **Function**:GO:0004523 (RNA-DNA hybrid ribonuclease activity), GO:0005524 (ATP binding), GO:0016740 (transferase activity), GO:0050567 (glutaminyl-tRNA synthase (glutamine-hydrolyzing) activity).
(*infB*) Translation initiation factor IF-2 †	HP1048	0.70 ± 0.04	**Process**:GO:0006184 (GTP catabolic process), GO:0006413 (translational initiation). **Function**:GO:0003743 (translation initiation factor activity), GO:0003924 (GTPase activity), GO:0005525 (GTP binding). **Component**:GO:0005737 (cytoplasm).
(*yidC*) Membrane protein insertase	HP1450	1.90 ± 0.04	**Process**:GO:0015031 (protein transport), GO:0051205 (protein insertion into membrane). **Component**: GO:0005886 (plasma membrane),GO:0016021 (integral component of membrane).

(*bamA*) Outer membrane protein assembly factor †	HP0655	1.55 ± 0.08	**Process**: GO:0006935 (chemotaxis), GO:0007165 (signal transduction), GO:0009454 (aerotaxis), GO:0043165 (Gram-negative-bacterium-type cell outer membrane assembly), GO:0050896 (response to stimulus), GO:0051205 (protein insertion into membrane). **Function**:GO:0004871 (signal transducer activity), GO:0005515 (protein binding), GO:0051082 (unfolded protein binding). **Component**: GO:0005886 (plasma membrane), GO:0009279 (cell outer membrane), GO:0016021 (integral to membrane).
(*secD*) Protein translocase subunit	HP1550	1.47 ± 0.05	**Process**: GO:0006605 (protein targeting), GO:0006886 (intracellular protein transport), GO:0043952 (protein transport by the Sec complex), GO:0046903 (secretion), GO:0065002 (intracellular protein transmembrane transport). **Function**: GO:0005886 (plasma membrane), GO:0015450 (P-P-bond-hydrolysis-driven protein transmembrane transporter activity). **Component**: GO:0016020 (membrane), GO:0016021 (integral to membrane).
(*clpX*) ATP-dependent Clp protease ATP-binding subunit †	HP1374	0.71 ± 0.03	**Process**:GO:0006457 (protein folding), GO:0006508 (proteolysis), GO:0006950 (response to stress). **Function**:GO:0003983 (UTP:glucose-1-phosphate uridylyltransferase activity), GO:0005524 (ATP binding), GO:0008233 (peptidase activity), GO:0008270 (zinc ion binding), GO:0017111 (nucleoside-triphosphatase activity), GO:0046872 (metal ion binding), GO:0046983 (protein dimerization activity), GO:0051082 (unfolded protein binding). **Component**:GO:0009368 (endopeptidase Clp complex)
(*tonB*) Protein tonB	HP1341	1.75 ± 0.03	**Process**:GO:0006810(transport),GO:0015031(protein transport), GO:0044718 (siderophore transmembrane transport). **Function**:GO:0015343(siderophore transmembrane transporter activity),GO:0031992(energy transducer activity). **Component**:GO:0005886 (plasma membrane), GO:0016021 (integral component of membrane), GO:0030288 (outer membrane-bounded periplasmic space).

(*secF*) Protein translocase subunit	HP1549	1.62 ± 0.09	**Process**:GO:0006605 (protein targeting),GO:0006886 (intracellular protein transport), GO:0043952 (protein transport by the Sec complex), GO:0046903 (secretion), GO:0065002 (intracellular protein transmembrane transport). **Function**:GO:0015450 (P-P-bond-hydrolysis-driven protein transmembrane transporter activity). **Component**: GO:0005886 (plasma membrane), GO:0016021 (integral to membrane).
(*tig*) Trigger factor †	HP0795	0.73 ± 0.01	**Process**:GO:0000413 (protein peptidyl-prolyl isomerization), GO:0006457 (protein folding), GO:0006461 (protein complex assembly), GO:0007049 (cell cycle), GO:0015031 (protein transport), GO:0043093 (FtsZ-dependent cytokinesis), GO:0051083 ('de novo' cotranslational protein folding), GO:0051301 (cell division). **Function**:GO:0003755 (peptidyl-prolyl cis-trans isomerase activity),GO:0005515 (protein binding),GO:0042802 (identical protein binding), GO:0043022 (ribosome binding), GO:0044183 (protein binding involved in protein folding). **Component**:GO:0005737 (cytoplasm), GO:0005829 (cytosol), GO:0016020 (membrane), GO:0044444 (cytoplasmic part).
Nucleic acid metabolic process			
(*HP0082*) Methyl-accepting chemotaxis transducer TlpC	HP0082	1.55 ± 0.07	**Process**:GO:0006259 (DNA metabolic process),GO:0006281 (DNA repair), GO:0007165 (signal transduction). **Function**:GO:0004871 (signal transducer activity),GO:0008725 (DNA-3-methyladenine glycosylase activity). **Component**: GO:0016020 (membrane).
(*greA*) Transcription elongation factor †	HP0866	0.48 ± 0.02	**Process**:GO:0006351 (transcription, DNA-dependent), GO:0006354 (DNA-templated transcription, elongation), GO:0006414 (translational elongation), GO:0016070 (RNA metabolic process), GO:0032784 (regulation of DNA-dependent transcription, elongation). **Function**:GO:0003677 (DNA binding), GO:0003746 (translation elongation factor activity).

(*ileS*) Isoleucine--tRNA ligase†	HP1422	0.71 ± 0.02	**Process**:GO:0006428 (isoleucyl-tRNAaminoacylation), GO:0006450 (regulation of translational fidelity). **Function**:GO:0002161 (aminoacyl-tRNA editing activity), GO:0004822 (isoleucine-tRNA ligase activity), GO:0005524 (ATP binding), GO:0008270 (zinc ion binding). **Component**:GO:0005737 (cytoplasm).
(*mnmG*) tRNA uridine 5-carboxymethylaminomethyl modification enzyme	HP0213	0.68 ± 0.01	**Process**:GO:0002098 (tRNA wobble uridine modification), GO:0030488 (tRNA methylation). **Function**:GO:0005515 (protein binding),GO:0050660 (flavin adenine dinucleotide binding). **Component**:GO:0005737 (cytoplasm).
(*nusG*) Transcription termination/antitermination protein	HP1203	0.74 ± 0.04	**Process**:GO:0006353(DNA-dependent transcription, termination), GO:0016070 (RNA metabolic process), GO:0031564 (transcription antitermination), GO:0032968 (positive regulation of transcription elongation from RNA polymerase II promoter).
(*polA*) DNA polymerase I †	HP1470	0.67 ± 0.10	**Process**:GO:0006260 (DNA replication), GO:0006281 (DNA repair), GO:0090305 (nucleic acid phosphodiester bond hydrolysis). **Function**:GO:0003677 (DNA binding), GO:0003887 (DNA-directed DNA polymerase activity), GO:0008408 (3'-5' exonuclease activity), GO:0008409 (5'-3' exonuclease activity).
(*alaS*) Alanine--tRNA ligase	HP1241	0.61 ± 0.05	**Process**:GO:0006419 (alanyl-tRNAaminoacylation). **Function**:GO:0000049 (tRNA binding), GO:0004813 (alanine-tRNA ligase activity), GO:0005524 (ATP binding), GO:0008270 (zinc ion binding). **Component**:GO:0005737 (cytoplasm).
(*HP0558*) 3-oxoacyl-[acyl-carrier-protein] synthase 2	HP0558	0.74 ± 0.02	**Process**:GO:0006633 (fatty acid biosynthetic process). **Function**:GO:0004315 (3-oxoacyl-[acyl-carrier-protein] synthase activity), GO:0033817 (beta-ketoacyl-acyl-carrier-protein synthase II activity).

(*rho*) Transcription termination factor Rho	HP0550	0.75 ± 0.02	**Process**:GO:0006353 (DNA-dependent transcription, termination), GO:0006355 (regulation of transcription, DNA-dependent), GO:0016070 (RNA metabolic process). **Function**:GO:0003723 (RNA binding), GO:0004386 (helicase activity), GO:0005524 (ATP binding), GO:0008186 (RNA-dependent ATPase activity).
(*HP0162*) Probable transcriptional regulatory protein	HP0162	0.69 ± 0.01	**Process**:GO:0006351 (transcription, DNA-dependent), GO:0006355 (regulation of transcription, DNA-dependent). **Function**:GO:0003677 (DNA binding). **Component**:GO:0005737 (cytoplasm).
ATP metabolic process			
(*HP0695*) Hydantoin utilization protein A HyuA	HP0695	0.37 ± 0.01	**Function**:GO:0016787 (hydrolase activity), GO:0047423 (N-methylhydantoinase (ATP-hydrolyzing) activity).
(*HP0696*) N-methylhydantoinase	HP0696	0.42 ± 0.03	**Process**:GO:0008152 (metabolic process). **Function**:GO:0003824 (catalytic activity), GO:0018710 (acetone carboxylase activity), GO:0047423 (N-methylhydantoinase (ATP-hydrolyzing) activity).
(*HP1137*) ATP synthase F0 sector subunit b' AtpF'	HP1137	1.65 ± 0.16	**Process**:GO:0015986(ATP synthesis coupled proton transport). **Function**:GO:0015078(hydrogen ion transmembrane transporter activity),GO:0016787(hydrolase activity). **Component**: GO:0016021 (integral component of membrane), GO:0045263 (proton-transporting ATP synthase complex, coupling factor F(o)).

(*atpD*) ATP synthase F1 sector subunit beta AtpD	HP1132	1.62 ± 0.02	**Process**:GO:0006091 (generation of precursor metabolites and energy), GO:0006754 (ATP biosynthetic process), GO:0006810 (transport), GO:0006811 (ion transport), GO:0015986 (ATP synthesis coupled proton transport), GO:0015991 (ATP hydrolysis coupled proton transport), GO:0015992 (proton transport), GO:0042777 (plasma membrane ATP synthesis coupled proton transport). **Function**:GO:0005524 (ATP binding), GO:0046933 (proton-transporting ATP synthase activity, rotational mechanism), GO:0046961 (proton-transporting ATPase activity, rotational mechanism). **Component**:GO:0005886 (plasma membrane), GO:0016021 (integral component of membrane), GO:0045261 (proton-transporting ATP synthase complex, catalytic core F(1)), GO:0045263 (proton-transporting ATP synthase complex, coupling factor F(o)), GO:0045264 (plasma membrane proton-transporting ATP synthase complex, coupling factor F(o)).
(*atpA*) ATP synthase F1 sector subunit alpha AtpA	HP1134	1.65 ± 0.10	**Process**:GO:0006091(generation of precursor metabolites and energy),GO:0015986(ATP synthesis coupled proton transport),GO:0015991(ATP hydrolysis coupled proton transport),GO:0042777(plasma membrane ATP synthesis coupled proton transport). **Function**:GO:0000166 (nucleotide binding),GO:0005515 (protein binding), GO:0005524 (ATP binding), GO:0016787 (hydrolase activity),GO:0016820 (hydrolase activity, acting on acid anhydrides, catalyzing transmembrane movement of substances), GO:0046933(proton-transporting ATP synthase activity, rotational mechanism), GO:0046961 (proton-transporting ATPase activity, rotational mechanism). **Component**: GO:0005886 (plasma membrane), GO:0016020 (membrane), GO:0045261 (proton-transporting ATP synthase complex, catalytic core F(1)), GO:0045262 (plasma membrane proton-transporting ATP synthase complex, catalytic core F(1)).
(*metN*) Methionine import ATP-binding protein MetN†	HP1576	1.42 ± 0.03	**Process**: GO:0006200 (ATP catabolic process), GO:0015821 (methionine transport). **Function**:GO:0005524 (ATP binding), GO:0016887 (ATPase activity), GO:0043865 (methionine transmembrane transporter activity). **Component**: GO:0005886 (plasma membrane).

(*atpG*) ATP synthase F1 sector gamma subunit AtpG†	HP1133	1.45 ± 0.08	**Process**:GO:0006091 (generation of precursor metabolites and energy),GO:0006259 (DNA metabolic process),GO:0006260 (DNA replication),GO:0006281 (DNA repair), GO:0006310 (DNA recombination), GO:0006754 (ATP biosynthetic process), GO:0006810 (transport), GO:0006811 (ion transport), GO:0015986 (ATP synthesis coupled proton transport),GO:0015992 (proton transport), GO:0042777 (plasma membrane ATP synthesis coupled proton transport). **Function**:GO:0005524 (ATP binding), GO:0046933 (proton-transporting ATP synthase activity, rotational mechanism), GO:0046961 (proton-transporting ATPase activity, rotational mechanism). **Component**: GO:0005886 (plasma membrane),GO:0016020 (membrane), GO:0045261 (proton-transporting ATP synthase complex, catalytic core F(1)), GO:0045262 (plasma membrane proton-transporting ATP synthase complex, catalytic core F(1)).
(*lon*) Lon protease †	HP1379	0.67 ± 0.02	**Process**:GO:0006200 (ATP catabolic process),GO:0006515 (misfolded or incompletely synthesized protein catabolic process), GO:0033554 (cellular response to stress). **Function**:GO:0004176 (ATP-dependent peptidase activity), GO:0004252 (serine-type endopeptidase activity), GO:0005524 (ATP binding), GO:0043565 (sequence-specific DNA binding). **Component**:GO:0005737 (cytoplasm).
(*HP1135*) ATP synthase F1 sector subunit delta AtpH	HP1135	1.54 ± 0.05	**Process**: GO:0015986 (ATP synthesis coupled proton transport). **Function**: GO:0046933 (proton-transporting ATP synthase activity, rotational mechanism). **Component**:GO:0005886 (plasma membrane), GO:0045261 (proton-transporting ATP synthase complex, catalytic core F(1)).
(*HP0569*) GTP-binding protein Gtp1	HP0569	0.47 ± 0.07	**Function**:GO:0005525 (GTP binding), GO:0016887 (ATPase activity).
Amino acid transport / metabolic process			
(*HP1399*) Arginase RocF	HP1399	0.66 ± 0.06	**Process**:GO:0006525 (arginine metabolic process). **Function**:GO:0004053 (arginase activity), GO:0046872 (metal ion binding).

(*dapD*) 2,3,4,5-tetrahydropyridine-2,6-dicarboxylate N-succinyltransferase	HP0626	0.54 ± 0.01	**Process**:GO:0009089 (lysine biosynthetic process via diaminopimelate), GO:0019877 (diaminopimelate biosynthetic process). **Function**:GO:0008666 (2,3,4,5-tetrahydropyridine-2,6-dicarboxylate N-succinyltransferase activity). **Component**:GO:0005737 (cytoplasm), GO:0009276 (Gram-negative-bacterium-type cell wall).
(*purA*) Adenylosuccinatesynthetase†	HP0255	0.55 ± 0.04	**Process**:GO:0006163 (purine nucleotide metabolic process), GO:0006164 (purine nucleotide biosynthetic process), GO:0006520 (cellular amino acid metabolic process), GO:0006522 (alanine metabolic process), GO:0006531 (aspartate metabolic process), GO:0006974 (cellular response to DNA damage stimulus), GO:0009117 (nucleotide metabolic process), GO:0009152 (purine ribonucleotide biosynthetic process), GO:0015949 (nucleobase-containing small molecule interconversion), GO:0044208 ('de novo' AMP biosynthetic process), GO:0046086 (adenosine biosynthetic process). **Function**:GO:0000166 (nucleotide binding), GO:0000287 (magnesium ion binding), GO:0004019 (adenylosuccinate synthase activity), GO:0004077 (biotin-[acetyl-CoA-carboxylase] ligase activity), GO:0005525 (GTP binding), GO:0046872 (metal ion binding). **Component**:GO:0005737 (cytoplasm), GO:0016020 (membrane).
(*hom*) Homoserine dehydrogenase†	HP0822	0.63 ± 0.03	**Process**:GO:0006520 (cellular amino acid metabolic process), GO:0006544 (glycine metabolic process), GO:0006563 (L-serine metabolic process), GO:0006566 (threonine metabolic process), GO:0009067 (aspartate family amino acid biosynthetic process), GO:0009085 (lysine biosynthetic process), GO:0009086 (methionine biosynthetic process), GO:0009088 (threonine biosynthetic process), GO:0009097 (isoleucine biosynthetic process), GO:0055114 (oxidation-reduction process). **Function**:GO:0004412 (homoserine dehydrogenase activity), GO:0016597 (amino acid binding), GO:0016620 (oxidoreductase activity, acting on the aldehyde or oxo group of donors, NAD or NADP as acceptor), GO:0050661 (NADP binding).
(*HP1118*) Gamma-glutamyltranspeptidaseGgt	HP1118	0.61 ± 0.02	**Process**:GO:0006749 (glutathione metabolic process). **Function**:GO:0003840 (gamma-glutamyltransferase activity).

(*HP0422*) Arginine decarboxylase SpeA	HP0422	1.46 ± 0.05	**Process**: GO:0006520 (cellular amino acid metabolic process), GO:0006527 (arginine catabolic process), GO:0006596 (polyamine biosynthetic process), GO:0008295 (spermidine biosynthetic process). **Function**: GO:0008792 (arginine decarboxylase activity).
(*HP1172*) Glutamine ABC transporter, periplasmic glutamine-binding protein GlnH	HP1172	1.73 ± 0.11	**Process**:GO:0006810(transport), GO:0006868 (glutamine transport). **Function**:GO:0005215 (transporter activity),GO:0047769(arogenate dehydratase activity). **Component**:GO:0042597(periplasmic space).
Phosphorylation			
(*ndk*) Nucleoside diphosphate kinase †	HP0198	0.40 ± 0.02	**Process**:GO:0006165 (nucleoside diphosphate phosphorylation), GO:0006183 (GTP biosynthetic process), GO:0006228 (UTP biosynthetic process), GO:0006241 (CTP biosynthetic process), GO:0009117 (nucleotide metabolic process), GO:0016310 (phosphorylation). **Function**:GO:0000166 (nucleotide binding), GO:0004519 (endonuclease activity), GO:0004550 (nucleoside diphosphate kinase activity), GO:0005515 (protein binding), GO:0005524 (ATP binding), GO:0042802 (identical protein binding), GO:0046872 (metal ion binding). **Component**:GO:0005737 (cytoplasm).
(*ppsA*) Phosphoenolpyruvate synthase †	HP0121	0.74 ± 0.03	**Process**:GO:0006090 (pyruvate metabolic process), GO:0006094 (gluconeogenesis), GO:0006259 (DNA metabolic process), GO:0006281 (DNA repair), GO:0006355 (regulation of transcription, DNA-templated), GO:0016310 (phosphorylation). **Function**:GO:0003908 (methylated-DNA-[protein]-cysteine S-methyltransferase activity), GO:0005524 (ATP binding), GO:0008986 (pyruvate, water dikinase activity), GO:0046872 (metal ion binding).

(*pgk*) Phosphoglycerate kinase	HP1345	0.67 ± 0.03	**Process**:GO:0006091 (generation of precursor metabolites and energy),GO:0006096 (glycolysis), GO:0016310 (phosphorylation), GO:0044248 (cellular catabolic process). **Function**:GO:0000166 (nucleotide binding), GO:0004618 (phosphoglycerate kinase activity), GO:0005524 (ATP binding), GO:0016301 (kinase activity), GO:0016740 (transferase activity). **Component**:GO:0005737 (cytoplasm).
(*HP1285*) Conserved hypothetical secreted protein	HP1285	0.55 ± 0.05	**Process**:GO:0016311 (dephosphorylation). **Function**:GO:0003993 (acid phosphatase activity). **Component**:GO:0009279 (cell outer membrane).
(*hldE*) Bifunctional proteinHldE†	HP0858	0.65 ± 0.03	**Process**:GO:0009244 (lipopolysaccharide core region biosynthetic process), GO:0046835 (carbohydrate phosphorylation), GO:0097171 (ADP-L-glycero-beta-D-manno-heptose biosynthetic process). **Funciton**:GO:0005524 (ATP binding), GO:0016773 (phosphotransferase activity, alcohol group as acceptor), GO:0016779 (nucleotidyltransferase activity), GO:0019200 (carbohydrate kinase activity).
Other (GTP, lipid, nucleoside, nucleotide, urea, cofactor) metabolic process			
(*HP0646*) UDP-glucose pyrophosphorylaseGalU	HP0646	0.66 ± 0.02	**Process**:GO:0006011 (UDP-glucose metabolic process), GO:0008152 (metabolic process), GO:0009058 (biosynthetic process), GO:0009225 (nucleotide-sugar metabolic process). **Function**:GO:0003983 (UTP:glucose-1-phosphate uridylyltransferase activity), GO:0008662 (1-phosphofructokinase activity).
(*apt*) Adenine phosphoribosyltransferase	HP0572	0.66 ± 0.04	**Process**:GO:0006166 (purine ribonucleoside salvage), GO:0006168 (adenine salvage), GO:0009116 (nucleoside metabolic process), GO:0044209 (AMP salvage). **Function**:GO:0000287 (magnesium ion binding),GO:0003999 (adenine phosphoribosyltransferase activity), GO:0042802 (identical protein binding). **Component**:GO:0005737 (cytoplasm), GO:0005829 (cytosol).

(*HP0690*) Acetyl coenzyme A acetyltransferase FadA†	HP0690	0.72 ± 0.11	**Process**:GO:0008152 (metabolic process), GO:0044255 (cellular lipid metabolic process). **Function**:GO:0003985 (acetyl-CoA C-acetyltransferase activity), GO:0008137 (NADH dehydrogenase (ubiquinone) activity), GO:0016747 (transferase activity, transferring acyl groups other than amino-acyl groups).
(*ureA*) Urease subunit alpha	HP0073	0.76 ± 0.01	**Process**:GO:0009405 (pathogenesis), GO:0043419 (urea catabolic process). **Function**: GO:0009039 (urease activity), GO:0016151 (nickel cation binding). **Component**:GO:0005737 (cytoplasm).
(*obg*) GTPaseobg†	HP0303	0.61 ± 0.01	**Process**:GO:0006184 (GTP catabolic process), GO:0050896 (response to stimulus). **Function**:GO:0000287 (magnesium ion binding), GO:0003924 (GTPase activity), GO:0005525 (GTP binding). **Component**:GO:0005737 (cytoplasm).
(*lpxA*) UDP-N-acetylglucosamine O-acyltransferase †	HP1375	0.51 ± 0.01	**Process**:GO:0006629 (lipid metabolic process), GO:0006935 (chemotaxis), GO:0007165 (signal transduction), GO:0008610 (lipid biosynthetic process), GO:0009245 (lipid A biosynthetic process), GO:0009454 (aerotaxis), GO:0050896 (response to stimulus). **Function**:GO:0004871 (signal transducer activity), GO:0008780 (acyl-[acyl-carrier-protein]-UDP-N-acetylglucosamine O-acyltransferase activity). **Component**:GO:0005737 (cytoplasm), GO:0009276 (Gram-negative-bacterium-type cell wall).
(*ureG*) Urease accessory protein	HP0068	0.61 ± 0.04	**Process**:GO:0006184 (GTP catabolic process), GO:0009405 (pathogenesis), GO:0019627 (urea metabolic process), GO:0051188 (cofactor biosynthetic process). **Function**:GO:0003924 (GTPase activity), GO:0005524 (ATP binding), GO:0005525 (GTP binding), GO:0016151 (nickel cation binding), GO:0016530 (metallochaperone activity), GO:0042803 (protein homodimerization activity), GO:0046872 (metal ion binding). **Component**:GO:0005737 (cytoplasm).

(*HP0654*) Putative uncharacterized protein	HP0654	0.66 ± 0.04	**Process**:GO:0009234 (menaquinone biosynthetic process), GO:0051188 (cofactor biosynthetic process). **Function**:GO:0016765 (transferase activity, transferring alkyl or aryl (other than methyl) groups), GO:0051539 (4 iron, 4 sulfur cluster binding).
Cell cycle			
(*ftsZ*) Cell division proteinFtsZ†	HP0979	0.68 ± 0.03	**Process**:GO:0000910 (cytokinesis),GO:0000917 (barrier septum assembly), GO:0006184 (GTP catabolic process), GO:0043093 (cytokinesis by binary fission), GO:0051258 (protein polymerization). **Function**:GO:0003924 (GTPase activity), GO:0005525 (GTP binding). **Component**:GO:0005737 (cytoplasm), GO:0032153 (cell division site), GO:0043234 (protein complex).
(*parB*) Probable chromosome-partitioning protein	HP1138	0.66 ± 0.02	**Process**:GO:0007059 (chromosome segregation). **Function**:GO:0003677 (DNA binding).
Response to stimulus			
(*HP0597*) Penicillin-binding protein 1A PBP-1A	HP0597	1.38 ± 0.01	**Process**:GO:0009252 (peptidoglycan biosynthetic process), GO:0046677 (response to antibiotic). **Function**:GO:0008233 (peptidase activity), GO:0008658 (penicillin binding), GO:0016763 (transferase activity, transferring pentosyl groups).
(*hcpC*) Putative beta-lactamase; Cysteine-rich protein C	HP1098	0.54 ± 0.13	**Process**:GO:0046677 (response to antibiotic). **Function**:GO:0008800 (beta-lactamase activity). **Component**:GO:0005576 (extracellular region).
CAG pathogenicity island protein			
(*cagA*) CAG pathogenicity island protein 26	HP0547	0.60 ± 0.01	**Process**:GO:1901998 (toxin transport). **Function**:GO:0019534 (toxin transporter activity).
(*HP0527*) CAG pathogenicity island protein 7 Cag7	HP0527	1.39 ± 0.01	

Process unknown			
(*lpp20*) LPP20 lipoprotein	HP1456	1.47 ± 0.12	**Component**: GO:0005886 (plasma membrane), GO:0009279 (cell outer membrane).
(*pgbB*) Plasminogen-binding protein	HP0863	1.36 ± 0.04	**Component**: GO:0009986 (cell surface).
(*HP1186*) Carbonic anhydrase	HP1186	0.54 ± 0.03	**Function**:GO:0004089 (carbonate dehydratase activity).
(*HP0231*) Uncharacterized protein	HP0231	0.63 ± 0.01	**Funciton**:GO:0016853 (isomerase activity).
(*HP0468*) Uncharacterized protein	HP0468	0.72 ± 0.03	**Function**:GO:0009055 (electron carrier activity), GO:0051536 (iron-sulfur cluster binding).
(*HP0492*) Uncharacterized protein	HP0492	1.96 ± 0.11	**Component**:GO:0009279 (cell outer membrane).
(*HP0697*) Uncharacterized protein	HP0697	0.40 ± 0.02	**Function**:GO:0018710 (acetone carboxylase activity).
(*HP1562*) Iron(III) ABC transporter, periplasmic iron-binding protein CeuE	HP1562	0.65 ± 0.03	**Function**: GO:0005506 (iron ion binding), GO:0046872 (metal ion binding).
(*HP0275*) ATP-dependent nuclease AddB	HP0275	0.73 ± 0.01	
(*HP0322*) Poly E-rich protein	HP0322	1.59 ± 0.11	
(*HP0596*) Tumor necrosis factor alpha-inducing protein	HP0596	1.45 ± 0.03	
(*HP1564*) Lipoprotein	HP1564	0.65 ± 0.06	

(*HP0025*) Outer membrane protein Omp2	HP0025	1.35 ± 0.04
(*HP0896*) Outer membrane protein Omp19	HP0896	1.41 ± 0.01
(*HP1177*) Outer membrane protein Omp27	HP1177	1.35 ± 0.04
(*HP0227*) Outer membrane protein Omp29	HP0227	1.47 ± 0.05
(*HP1286*) Conserved hypothetical secreted protein	HP1286	0.54 ± 0.03
(*HP0014*) Uncharacterized protein	HP0014	0.46 ± 0.03
(*HP0185*) Uncharacterized protein	HP0185	1.83 ± 0.21
(*HP0305*) Uncharacterized protein	HP0305	0.65 ± 0.09
(*HP0385*) Uncharacterized protein	HP0385	1.50 ± 0.09
(*HP0406*) Uncharacterized protein	HP0406	1.74 ± 0.17
(*HP0466*) Uncharacterized protein	HP0466	1.57 ± 0.13
(*HP0721*) Uncharacterized protein	HP0721	0.50 ± 0.02
(*HP0746*) Uncharacterized protein	HP0746	1.40 ± 0.05
(*HP0953*) Uncharacterized protein	HP0953	0.60 ± 0.05
(*HP0958*) Uncharacterized protein	HP0958	0.60 ± 0.05

(*HP1173*) Uncharacterized protein	HP1173	0.59 ± 0.04
(*HP1236*) Uncharacterized protein	HP1236	0.58 ± 0.01
(*HP1334*) Uncharacterized protein	HP1334	0.73 ± 0.01

† Proteins involved in more than one biological process categories based on current classification criteria. **CcoP**: Oxidation-reduction process, Phosphorylation, ATP metabolic process; **KatA**: Oxidation-reduction process, Response to stimulus; **Tpx**: Oxidation-reduction process, Response to stimulus; **AcsA**: Oxidation-reduction process, Cofactor metabolic process; **HP0318**: Oxidation-reduction process, Cofactor metabolic process; **HspB**: Oxidation-reduction process, Protein metabolic process, Response to stimulus; **TufA**: Protein metabolic process, GTP metabolic process; **MetG**: Protein metabolic process, Nucleic acid metabolic process; **GatA**: Protein metabolic process, Nucleic acid metabolic process; **InfB**: Protein metabolic process, GTP metabolic process; **BamA**: Protein transport, Response to stimulus; **ClpX**: Protein metabolic process, Response to stimulus; **Tig**: Protein transport / metabolic process, Cell cycle; **GreA**: Nucleic acid metabolic process, Protein metabolic process; **IleS**: Nucleic acid metabolic process, Protein metabolic process; **PolA**: Nucleic acid metabolic process, Response to stimulus; **MetN**: ATP metabolic process, Amino acid transport; **AtpG**: ATP metabolic process, Nucleic acid metabolic process; **Lon**: ATP metabolic process, Protein metabolic process, Response to stimulus; **PurA**: Amino acid metabolic process, Nucleotide metabolic process, Response to stimulus; **Hom**: Amino acid metabolic process, Oxidation-reduction process; **Ndk**: Phosphorylation, GTP and nucleotide metabolic process; **PpsA**: Phosphorylation, Nucleic acid metabolic process; **HldE**: Phosphorylation, Lipid and nucleotide metabolic process; **FadA**: Lipid metabolic process, Oxidation-reduction process; **Obg**: GTP metabolic process, response to stimulus; **LpxA**: Lipid metabolic process, Response to stimulus; **FtsZ**: Cell cycle, GTP metabolic process.

Table S4 Enriched functional PPI subnetworks associated with the Bi-influenced proteins in *H. pylori*.

**Subnetwork**	**GO ID**	***p*-value**	**FDR**	**Regulation** [†]	***p*-value** [†]	**FDR** [†]	**Protein Ratio** [‡]
tricarboxylic acid cycle	GO:0006099	1.05×10^{-9}	1.22×10^{-7}	down	3.28×10^{-7}	2.55×10^{-5}	32/110
cell redox homeostasis	GO:0045454	4.44×10^{-9}	2.60×10^{-7}	down	3.02×10^{-5}	5.74×10^{-4}	20/49
Ni homeostasis [*]	–	6.93×10^{-9}	2.95×10^{-7}	down	9.75×10^{-3}	3.66×10^{-2}	51/248
response to oxidative stress	GO:0006979	7.57×10^{-9}	2.95×10^{-7}	down	8.50×10^{-4}	6.39×10^{-3}	22/59
protein folding	GO:0006457	1.95×10^{-8}	5.71×10^{-7}	down	3.20×10^{-5}	5.74×10^{-4}	43/197
Fe homeostasis [*]	–	6.65×10^{-8}	1.60×10^{-6}	down	3.27×10^{-4}	3.18×10^{-3}	83/543
electron transport chain	GO:0022900	1.99×10^{-7}	3.33×10^{-6}	down	3.87×10^{-3}	1.84×10^{-2}	31/127
protein complex assembly	GO:0006461	2.48×10^{-7}	3.62×10^{-6}	down	6.80×10^{-4}	5.28×10^{-3}	37/167
oxidation-reduction process	GO:0055114	4.12×10^{-7}	5.68×10^{-6}	down	5.78×10^{-6}	2.25×10^{-4}	96/690
proteolysis	GO:0006508	5.66×10^{-7}	7.36×10^{-6}	down	5.85×10^{-5}	9.09×10^{-4}	57/328
urea metabolic process	GO:0019627	2.23×10^{-6}	2.37×10^{-5}	down	1.03×10^{-2}	3.78×10^{-2}	23/88
plasma membrane ATP synthesis coupled proton transport	GO:0042777	1.28×10^{-5}	9.39×10^{-5}	up	7.49×10^{-5}	8.73×10^{-3}	16/52
RNA metabolic process	GO:0016070	2.56×10^{-5}	1.66×10^{-4}	down	1.39×10^{-3}	8.29×10^{-3}	99/770
translational elongation	GO:0006414	4.01×10^{-5}	2.47×10^{-4}	down	8.78×10^{-4}	6.39×10^{-3}	29/144
GTP catabolic process	GO:0006184	2.51×10^{-4}	1.15×10^{-3}	down	1.04×10^{-2}	3.78×10^{-2}	38/233

pathogenesis	GO:0009405	2.99×10^{-4}	1.29×10^{-3}	–	–	–	34/206
ATP metabolic process	GO:0046034	5.31×10^{-4}	1.87×10^{-3}	–	–	–	40/260

† Regulation was determined by calculating the enrichment *p*-values and FDR values of the up- or down-regulated proteins in each functional subnetworks.

‡ Numberof Bi-influenced proteins / total number of proteins in each functional subnetwork

* Proteins involving in Ni/Fe homeostasis were collected from the reported nickel homeostasis[17-21] or iron homeostasis[18, 22] proteins in *H. pylori*, as well as the proteins annotated by nickel- or iron-related GO terms.

Table S5 Bi-associated *H. pylori* proteins annotated with catalytic functions.

**Locus tag**[*]	**Gene name**	**Catalytic functions**
**HP0010**	*groEL*	GO:0016491 (oxidoreductase activity)
**HP0027**	*icd*	GO:0004450 (isocitrate dehydrogenase (NADP+) activity); GO:0016491 (oxidoreductase activity)
**HP0068**	*ureG*	GO:0003924 (GTPase activity); GO:0016787 (hydrolase activity)
**HP0072**	*ureB*	GO:0009039 (urease activity); GO:0016787 (hydrolase activity)
**HP0073**	*ureA*	GO:0009039 (urease activity); GO:0016787 (hydrolase activity)
HP0082	*HP0082*	GO:0008725 (DNA-3-methyl adenine glycosylase activity); GO:0016787 (hydrolase activity)
HP0121	*ppsA*	GO:0003908 (methylated-DNA-[protein]-cysteine S-methyltransferase activity); GO:0008986 (pyruvate, water dikinase activity); GO:0016740 (transferase activity)
HP0136	*bcp*	GO:0003908 (methylated-DNA-[protein]-cysteine S-methyltransferase activity); GO:0008986 (pyruvate, water dikinase activity); GO:0016740 (transferase activity); GO:0016491 (oxidoreductase activity)
**HP0138**	*HP0138*	GO:0016491 (oxidoreductase activity)
HP0147	*ccoP*	GO:0016491 (oxidoreductase activity)
**HP0154**	*eno*	GO:0004634 (phosphopyruvate hydratase activity); GO:0016829 (lyase activity)
**HP0175**	*HP0175*	GO:0003755 (peptidyl-prolyl cis-trans isomerase activity); GO:0016853 (isomerase activity); GO:0008233 (peptidase activity); GO:0016787 (hydrolase activity)
**HP0176**	*fba*	GO:0016832 (aldehyde-lyase activity); GO:0009025 (tagatose-bisphosphate aldolase activity); GO:0004332 (fructose-bisphosphate aldolase activity); GO:0016829 (lyase activity)
**HP0191**	*frdB*	GO:0000104 (succinate dehydrogenase actiivty); GO:0016491 (oxidoreductase activity)
**HP0192**	*frdA*	GO:0000104 (succinate dehydrogenase actiivty); GO:0016491 (oxidoreductase activity)
**HP0197**	*metK*	GO:0004478 (methionine adenosyltransferase activity); GO:0004713 (protein tyrosine kinase activity); GO:0016740 (transferase activity)

HP0198	*ndk*	GO:0004550 (nucleoside diphosphate kinase activity); GO:0016740 (transferase activity); GO:0004519 (endonuclease activity); GO:0016787 (hydrolase activity)
**HP0202**	*fabH*	GO:0004315 (3-oxoacyl-[acyl-carrier-protein] synthase activity); GO:0033818 (beta-ketoacyl-acyl-carrier-protein synthase III activity); GO:0016740 (transferase activity)
**HP0210**	*htpG*	GO:0016407 (acetyltransferase activity); GO:0016740 (transferase activity); GO:0042623 (ATPase activity, coupled); GO:0016787 (hydrolase activity)
HP0231	*HP0231*	GO:0016853 (isomerase activity)
**HP0243**	*napA*	GO:0016722 (oxidoreductase activity, oxidizing metal ions); GO:0016491 (oxidoreductase activity)
HP0255	*purA*	GO:0004077 (biotin-[acetyl-CoA-carboxylase] ligase activity); GO:0004019 (adenylosuccinate synthase activity); GO:0016874 (ligase activity)
**HP0294**	*amiE*	GO:0004040 (amidase activity); GO:0016787 (hydrolase activity)
HP0303	*obg*	GO:0003924 (GTPase activity); GO:0016787 (hydrolase activity)
HP0318	*HP0318*	GO:0004733 (pyridoxamine-phosphate oxidase activity); GO:0016491 (oxidoreductase activity)
HP0390	*tpx*	GO:0008379 (thioredoxin peroxidase activity); GO:0016491 (oxidoreductase activity)
HP0417	*metG*	GO:0008463 (formylmethioninedeformylase activity); GO:0016787 (hydrolase activity); GO:0004825 (methionine-tRNA ligase activity); GO:0016874 (ligase activity)
HP0422	*HP0422*	GO:0008792 (arginine decarboxylase activity); GO:0016829 (lyase activity)
**HP0480**	*typA*	GO:0003924 (GTPase activity); GO:0016787 (hydrolase activity)
**HP0512**	*glnA*	GO:0004356 (glutamate-ammonia ligase activity); GO:0016874 (ligase activity)
HP0550	*rho*	GO:0008186 (RNA-dependent ATPase activity); GO:0004386 (helicase activity); GO:0016787 (hydrolase activity)
**HP0557**	*accA*	GO:0003989 (acetyl-CoA carboxylase activity); GO:0016874 (ligase activity); GO:0004810 (tRNAadenylyltransferase activity); GO:0052929 (ATP:3'-cytidine-cytidine-tRNA adenylyltransferase activity); GO:0052928 (CTP:3'-cytidine-tRNA cytidylyltransferase activity); GO:0052927 (CTP:tRNAcytidylyltransferase activity); GO:0016437 (tRNAcytidylyltransferase activity); GO:0016740 (transferase activity); GO:0008081 (phosphoric diester hydrolase activity); GO:0016787 (hydrolase activity)

HP0558	*HP0558*	GO:0004315 (3-oxoacyl-[acyl-carrier-protein] synthase activity); GO:0033817 (beta-ketoacyl-acyl-carrier-protein synthase II activity); GO:0016740 (transferase activity)
HP0572	*apt*	GO:0003999 (adenine phosphoribosyltransferase activity); GO:0016740 (transferase activity)
HP0588	*HP0588*	GO:0047553 (2-oxoglutarate synthase activity); GO:0016491 (oxidoreductase activity)
**HP0589**	*HP0589*	GO:0047553 (2-oxoglutarate synthase activity); GO:0016491 (oxidoreductase activity)
HP0590	*HP0590*	GO:0047553 (2-oxoglutarate synthase activity); GO:0016491 (oxidoreductase activity)
HP0591	*HP0591*	GO:0016903 (oxidoreductase activity, acting on the aldehyde or oxo group of donors); GO:0016491 (oxidoreductase activity)
HP0597	*HP0597*	GO:0008233 (peptidase activity); GO:0016787 (hydrolase activity); GO:0016763 (transferase activity, transferring pentosyl groups); GO:0016740 (transferase activity)
**HP0620**	*ppa*	GO:0004427 (inorganic diphosphatase activity); GO:0016787 (hydrolase activity)
HP0626	*dapD*	GO:0008666 (2,3,4,5-tetrahydropyridine-2,6-dicarboxylate N-succinyltransferase activity); GO:0016740 (transferase activity)
**HP0631**	*HP0631*	GO:0047067 (hydrogen:quinone oxidoreductase activity); GO:0008901 (ferredoxin hydrogenase activity); GO:0016491 (oxidoreductase activity)
HP0632	*HP0632*	GO:0047067 (hydrogen:quinone oxidoreductase activity); GO:0008901 (ferredoxin hydrogenase activity); GO:0016491 (oxidoreductase activity)
HP0646	*HP0646*	GO:0008662 (1-phosphofructokinase activity); GO:0003983 (UTP:glucose-1-phosphate uridylyltransferase activity); GO:0016740 (transferase activity)
**HP0653**	*ftnA*	GO:0004322 (ferroxidase activity); GO:0016491 (oxidoreductase activity)
HP0654	*HP0654*	GO:0016765 (transferase activity, transferring alkyl or aryl (other than methyl) groups); GO:0016740 (transferase activity)
HP0690	*HP0690*	GO:0008137 (NADH dehydrogenase (ubiquinone) activity); GO:0016491 (oxidoreductase activity); GO:0003985 (acetyl-CoA C-acetyltransferase activity); GO:0016747 (transferase activity, transferring acyl groups other than amino-acyl groups); GO:0016740 (transferase activity)

HP0691	*scoA*	GO:0004318 (enoyl-[acyl-carrier-protein] reductase (NADH) activity); GO:0008260 (3-oxoacid CoA-transferase activity); GO:0016740 (transferase activity); GO:0004450 (isocitrate dehydrogenase (NADP+) activity); GO:0016491 (oxidoreductase activity)
HP0692	*scoB*	GO:0008260 (3-oxoacid CoA-transferase activity); GO:0016740 (transferase activity)
HP0695	*HP0695*	GO:0047423 (N-methylhydantoinase (ATP-hydrolyzing) activity); GO:0016787 (hydrolase activity)
HP0696	*HP0696*	GO:0018710 (acetone carboxylase activity); GO:0016874 (ligase activity); GO:0047423 (N-methylhydantoinase (ATP-hydrolyzing) activity); GO:0016787 (hydrolase activity)
**HP0697**	*HP0697*	GO:0018710 (acetone carboxylase activity); GO:0016874 (ligase activity)
**HP0779**	*acnB*	GO:0047456 (2-methylisocitrate dehydratase activity); GO:0047780 (citrate dehydratase activity); GO:0052633 (isocitrate hydro-lyase (cis-aconitate-forming) activity); GO:0016829 (lyase activity)
**HP0795**	*tig*	GO:0003755 (peptidyl-prolyl cis-trans isomerase activity); GO:0016853 (isomerase activity)
HP0822	*hom*	GO:0016620 (oxidoreductase activity, acting on the aldehyde or oxo group of donors, NAD or NADP as scceptor); GO:0004412 (homoserine dehydrogenase activity); GO:0016491 (oxidoreductase activity)
**HP0824**	*trxA*	GO:0015035 (protein disulfide oxidoreductase activity); GO:0016491 (oxidoreductase activity)
**HP0825**	*trxB*	GO:0004791 (thioredoxin-disulfide reductase activity); GO:0016491 (oxidoreductase activity)
HP0830	*gatA*	GO:0004523 (RNA-DNA hybrid ribonuclease activity); GO:0016787 (hydrolase activity); GO:0050567 (glutaminyl-tRNA synthase (glutamine-hydrolyzing) activity); GO:0016874 (ligase activity); GO:0016740 (transferase activity)
HP0858	*hldE*	GO:0016779 (nucleotihyltransferase activity); GO:0016773 (phosphotransferase activity, alcohol group as acceptor); GO:0019200 (carbohydrate kinase activity); GO:0016740 (transferase activity)
**HP0875**	*katA*	GO:0016407 (acetyltransferase activity); GO:0016740 (transferase activity); GO:0004096 (catalase activity); GO:0016491 (oxidoreductase activity)
**HP0900**	*hypB*	GO:0003924 (GTPase activity); GO:0016787 (hydrolase activity)
HP0977	*HP0977*	GO:0003755 (peptidyl-prolyl cis-trans isomerase activity); GO:0016853 (isomerase activity)
HP0979	*ftsZ*	GO:0003924 (GTPase activity); GO:0016787 (hydrolase activity)

**HP1037**	*HP1037*	GO:0016787 (hydrolase activity)
HP1045	*acsA*	GO:0004823 (leucine-tRNA ligase activity); GO:0003987 (acetate-CoA ligase activity); GO:0016874 (ligase activity)
HP1048	*infB*	GO:0003924 (GTPase activity); GO:0016787 (hydrolase activity)
HP1098	*hcpC*	GO:0008800 (beta-lactamase activity); GO:0016787 (hydrolase activity)
HP1108	*HP1108*	GO:0019164 (pyruvate synthase activity); GO:0016625 (oxidoreductase activity, acting on the aldehyde or oxo group of donors, iron-sulfur protein as acceptor); GO:0016491 (oxidoreductase activity)
HP1109	*HP1109*	GO:0019164 (pyruvate synthase activity); GO:0016625 (oxidoreductase activity, acting on the aldehyde or oxo group of donors, iron-sulfur protein as acceptor); GO:0016491 (oxidoreductase activity)
HP1110	*HP1110*	GO:0019164 (pyruvate synthase activity); GO:0016491 (oxidoreductase activity)
**HP1111**	*HP1111*	GO:0019164 (pyruvate synthase activity); GO:0016491 (oxidoreductase activity)
**HP1118**	*HP1118*	GO:0003840 (gamma-glutamyltransferase activity); GO:0016740 (transferase activity)
**HP1132**	*atpD*	GO:0046933 (proton-transporting ATP synthase activity, rotational mechanism); GO:0046961 (proton-transporting ATPase activity, rotational mechanism); GO:0016787 (hydrolase activity)
HP1133	*atpG*	GO:0046933 (proton-transporting ATP synthase activity, rotational mechanism); GO:0046961 (proton-transporting ATPase activity, rotational mechanism); GO:0016787 (hydrolase activity)
**HP1134**	*atpA*	GO:0046933 (proton-transporting ATP synthase activity, rotational mechanism); GO:0046961 (proton-transporting ATPase activity, rotational mechanism); GO:0016820 (hydrolase activity, acting on acid anhydrides, catalyzing transmembrane movement of substances); GO:0016787 (hydrolase activity)
HP1135	*HP1135*	GO:0046933 (proton-transporting ATP synthase activity, rotational mechanism); GO:0016787 (hydrolase activity)
HP1137	*HP1137*	GO:0016787 (hydrolase activity)
**HP1161**	*fldA*	GO:0016491 (oxidoreductase activity)
HP1172	*HP1172*	GO:0047769 (arogenate dehydratase activity); GO:0016829 (lyase activity)
HP1186	*HP1186*	GO:0004089 (carbonate dehydratase activity); GO:0016829 (lyase activity)
**HP1195**	*fusA*	GO:0003924 (GTPase activity); GO:0016787 (hydrolase activity)

**HP1205**	*tuf*	GO:0003924 (GTPase activity); GO:0016787 (hydrolase activity)
HP1241	*alaS*	GO:0004813 (alanine-tRNA ligase activity); GO:0016874 (ligase activity)
HP1285	*HP1285*	GO:0003993 (acid phosphatase activity); GO:0016787 (hydrolase activity)
**HP1293**	*rpoA*	GO:0003899 (DNA-directed RNA polymerase activity); GO:0016740 (transferase activity)
**HP1325**	*fumC*	GO:0004333 (fumarate hydratase activity); GO:0016829 (lyase activity)
HP1345	*pgk*	GO:0004618 (phosphoglycerate kinase activity); GO:0016301 (kinase activity); GO:0016740 (transferase activity)
HP1374	*clpX*	GO:0003983 (UTP:glucose-1-phosphate uridylyltransferase activity); GO:0016740 (transferase activity); GO:0017111 (nucleoside-triphosphatase activity); GO:0008233 (peptidase activity); GO:0016787 (hydrolase activity)
HP1375	*lpxA*	GO:0008780 (acyl-[acyl-carrier-protein]-UDP-N-acetylglucosamine O-acyltransferase activity); GO:0016740 (transferase activity)
HP1379	*lon*	GO:0016887 (ATPase activity); GO:0004176 (ATP-dependent peptidase activity); GO:0004252 (serine-type endopeptidase activity); GO:0016787 (hydrolase activity)
HP1399	*HP1399*	GO:0004053 (arginase activity); GO:0016787 (hydrolase activity)
HP1422	*ileS*	GO:0004822 (isoleucine-tRNA ligase activity); GO:0016874 (ligase activity)
HP1441	*ppiA*	GO:0003755 (peptidyl-prolyl cis-trans isomerase activity); GO:0016853 (isomerase activity)
HP1458	*HP1458*	GO:0015035 (protein disulfide oxidoreductase activity); GO:0016491 (oxidoreductase activity)
HP1470	*polA*	GO:0003887 (DNA-directed DNA polymerase activity); GO:0016740 (transferase activity); GO:0008408 (3'-5' exonuclease activity); GO:0008409 (5'-3' exonuclease activity); GO:0016787 (hydrolase activity)
HP1507	*HP1507*	GO:0004754 (saccharopine dehydrogenase (NAD+, L-lysine-forming) activity); GO:0016491 (oxidoreductase activity)
**HP1563**	*tsaA*	GO:0004601 (peroxidase activity); GO:0016491 (oxidoreductase activity)
HP1576	*metN*	GO:0016887 (ATPase activity); GO:0016787 (hydrolase activity)

* Bi-binding proteins are highlighted in bold. The down- and up-regulated proteins induced by bismuth are single and double underlined, respectively.

Table S6 Statistical analysis of Bi-influenced catalytic functions.

**GO ID**	**GO term name**	**Bi-protein Ratio**	**Background Ratio**	***p*-value**	**FDR**
GO:0016491	oxidoreductase activity	31/153	125/1555	5.74×10^{-7}	3.44×10^{-6}
GO:0016787	hydrolase activity	37/153	296/1555	2.01×10^{-2}	6.04×10^{-2}
GO:0016874	ligase activity	10/153	64/1555	4.70×10^{-2}	9.40×10^{-2}
GO:0016829	lyase activity	7/153	67/1555	0.16	0.22
GO:0016853	isomerase activity	5/153	49/1555	0.19	0.22
GO:0016740	transferase activity	24/153	269/1555	0.92	0.92

Table S7 Network parameters of *H. pylori* protein-protein interaction networks.

Network	Number of nodes	Number of edges	Proteome connectivity	α value	Average shortest path	Average degree	Degree centrality	Betweenness centrality	Closeness centrality	Clustering coefficient
HPI	1520	11350	0.9775	2.37	3.45	14.93	0.0495	0.0223	2.65×10^{-6}	0.4439
BiPI	967	2636	0.6219	2.51	4.21	5.45	0.0701	0.0953	2.47×10^{-4}	0.1693
Core-BiPI	130	390	0.0836	2.10	3.92	6.00	0.133	0.1526	2.14×10^{-4}	0.4174

Table S8 List of proteins in BiPI network with a large degree or BC value, and their CC values. [*]

Top degree nodes	Degree	CC ($\times 10^{-4}$)	Top BC nodes	BC	CC ($\times 10^{-4}$)
**HP1293**	73	3.5574	**HP0109**	45965.45	3.7425
HP1195	72	3.4578	**HP0073**	38218.26	3.6456
HP1312	67	3.2563	**HP0072**	33906.07	3.6603
HP1554	66	3.2616	HP0010	31713.37	3.7879
**HP1203**	64	3.3080	HP0512	28997.71	3.3933
**HP0109**	62	3.7425	**HP1203**	26284.48	3.5348
**HP0073**	62	3.6456	HP1134	25376.76	3.3967
HP0795	61	3.4048	HP0547	24991.70	3.6166
**HP0072**	58	3.5855	**HP1293**	23567.25	3.4376
HP1205	55	3.4282	HP0979	19021.21	3.3378
HP0010	51	3.7120	HP0198	17975.63	3.0202
HP0547	51	3.2862	HP1135	16967.55	3.1526
HP0399	49	3.1260	HP1285	16070.05	3.5373
HP1555	48	3.2310	HP1195	15827.17	3.2300
HP1048	47	3.0367	HP1554	15596.25	2.8241
HP1134	44	3.4638	HP0958	14757.84	3.1476
HP0512	42	3.5549	HP0887	14651.52	3.5411
HP0527	39	2.7701	HP0197	14635.82	3.2819
HP0198	34	3.1211	HP1118	14510.93	3.0988
HP0979	33	3.2584	HP0136	14362.38	3.1867
HP0875	33	3.4223	HP0655	14318.76	3.3367
HP0154	31	3.2289	HP0795	14049.46	3.5511
HP1563	30	3.4329	HP0597	13363.42	3.0912
HP0588	29	2.9446	HP1205	13166.84	2.7972
HP0191	28	3.1348	HP1563	13133.2	3.0175
HP1118	28	3.2072	HP0721	13096.54	3.5298
HP1135	28	3.1596	HP0191	13008.61	3.4590
HP1285	27	2.8769	HP0875	12925.06	3.3591
HP0177	27	3.0166	HP1312	12834.96	2.9833
HP0653	26	2.1250	HP0954	12593.49	2.9231
HP0824	25	2.3534	HP1422	12406.27	3.0788
HP0958	25	2.8114	HP0822	11991.02	2.7012

HP1550	25	2.8177	HP0953	11877.81	3.1766
HP1109	25	2.9507	HP0154	11774.05	3.2154
HP1111	25	2.9533	HP0026	11417.55	3.1037
HP0011	24	3.2446	HP0399	11266.91	2.9594
HP0822	24	2.9257	HP0824	11085.10	3.4459
HP1549	24	2.8050	HP0243	11042.68	3.5311
HP1399	23	3.1446	HP0692	10255.42	3.0230
HP0176	23	3.0175	HP0406	10157.27	3.0779
HP1422	23	2.9833	HP0588	10052.72	2.8902
HP0202	23	2.9087	HP0468	9540.71	3.1172
HP0721	22	2.6976	HP0866	9287.09	3.1008
HP0620	22	2.9586	HP0527	9188.67	2.9070
HP0197	22	3.1827	HP1456	9115.81	3.2862
HP1132	22	3.1706	HP1550	8911.31	2.8161
HP1110	22	2.8769	HP0175	8903.08	2.9464
HP0597	22	2.8818	HP1198	8734.59	3.4118
HP0655	22	2.9833	HP0255	8553.88	2.4925

* Proteins with both large degree and BC values (top 5% of the total nodes in the network) are highlighted underlined. Proteins highlighted in bold represent the most central nodes in the BiPI network (nodes with both large degree and BC value within the 1% of the total nodes in the network).

Table S9 Differential characteristics averaged for hubs, non-hubs and Bi-binding proteins in the BiPI network.

	**hubs**	**non-hubs**	**Bi-binding proteins**	***p*-values*** **(hubs *vs* non-hubs)**	***p*-values*** **(hubs *vs* Bi-binding proteins)**	***p*-values*** **(non-hubs *vs* Bi-binding proteins)**
Number of proteins	97	871	63	NA	NA	NA
Average network degree	27.04	3.06	24.46	0.00	0.026	1.00
Average network betweenness	10988.74	505.69	9484.61	0.00	0.001	1.00

**p*-values from two sample Wilcoxon Signed Rank Test.

References

1. L. Hu, T. Cheng, B. He, L. Li, Y. Wang, Y. T. Lai, G. Jiang and H. Sun, *Angew. Chem. Int. Ed.*, 2013, **52**, 4916-4920.
2. J. R. Wisniewski, A. Zougman, N. Nagaraj and M. Mann, *Nat. Methods*, 2009, **6**, 359-362.
3. Q. Quan, S. S. Szeto, H. C. Law, Z. Zhang, Y. Wang and I. K. Chu, *Anal. Chem.*, 2015, **87**, 10015-10024.
4. D. M. Molina, R. Jafari, M. Ignatushchenko, T. Seki, E. A. Larsson, C. Dan, L. Sreekumar, Y. Cao and P. Nordlund, *Science*, 2013, **341**, 84-87.
5. J.-C. R. Rain, L. Selig, H. D. Reuse, V. Battaglia, C. Reverdy, S. Simon, G. Lenzen, F. Petel, J. Wojcik, V. Schachter, Y. Chemama, A. Labigne and P. Legrain, *Nature*, 2001, **409**, 211-215.
6. R. Häuser, A. Ceol, S. V. Rajagopala, R. Mosca, G. Siszler, N. Wermke, P. Sikorski, F. Schwarz, M. Schick, S. Wuchty, P. Aloy and P. Uetz, *Mol. Cell. Proteomics*, 2014, **13**, 1318-1329.
7. M. W. Weatherburn, *Anal. Chem.*, 1967, **39**, 971-974.
8. T. Peng and D. Yang, *Org. Lett.*, 2010, **12**, 4932-4935.
9. K. J. Nelson and D. Parsonage, *Curr. Protoc. Toxicol.*, 2011, **Unit 7.10**, 7.10.11-17.10.28.
10. N. Shangari and P. J. O'Brien, *Curr. Protoc. Toxicol.*, 2006, **Unit 7.7**, 7.7.1-7.7.16.
11. D. R. Spitz and L. W. Oberley, *Curr. Protoc. Toxicol.*, 2001, **Unit 7.5**, 7.5.1-7.5.11.
12. D. J. McGee, J. Zabaleta, R. J. Viator, T. L. Testerman, A. C. Ochoa and G. L. Mendz, *Eur. J. Biochem.*, 2004, **271**, 1952-1962.
13. J. Buchner, H. Grallert and U. Jakob, in *Methods in Enzymology*, 1998, vol. 290, ch. Molecular Chaperones, pp. 323-338.
14. D. Mamelak and C. Lingwood, *J. Biol. Chem.*, 2001, **276**, 449-456.
15. R. Ge, X. Sun, Q. Gu, R. M. Watt, J. A. Tanner, B. C. Wong, H. H. Xia, J. D. Huang, Q. Y. He and H. Sun, *J. Biol. Inorg. Chem.*, 2007, **12**, 831-842.
16. Y. Wang, C. N. Tsang, F. Xu, P. W. Kong, L. Hu, J. Wang, I. K. Chu, H. Li and H. Sun, *Chem. Commun.*, 2015, **51**, 16479-16482.
17. X. Yang, D. Xu, T. Cheng, Z. Xi, L. Zhao, Y. Liu and H. Sun, *Prog. Chem.*, 2013, **25**, 495-510.
18. A. Danielli and V. Scarlato, *FEMS Microbiol. Rev.*, 2010, **34**, 738-752.
19. K. Schauer, B. Gouget, M. Carriere, A. Labigne and H. de Reuse, *Mol. Microbiol.*, 2007, **63**, 1054-1068.
20. F. N. Stahler, S. Odenbreit, R. Haas, J. Wilrich, A. H. Van Vliet, J. G. Kusters, M. Kist and S. Bereswill, *Infect. Immun.*, 2006, **74**, 3845-3852.
21. R. J. Maier, S. L. Benoit and S. Seshadri, *Biometals*, 2007, **20**, 655-664.
22. R. Ge and X. Sun, *Biometals*, 2012, **25**, 247-258.
